# Modelling future dental workforce need for adults with special care needs within the UK

**DOI:** 10.1038/s41415-026-0038-4

**Published:** 2026-11-13

**Authors:** Cyonee Mukhopadhyaya, Jalpa Patel, Charlotte Curl, Paul R. Harper, Jennifer E. Gallagher

**Affiliations:** 331706786691912608866 School of Mathematics, Cardiff University, Senghennydd Road, Cardiff, CF24 4AG, United Kingdom; 029234762590252020078 Special Care Dentistry, King´s College Hospital, Bessemer Road, Denmark Hill, SE5 9RW, United Kingdom; 718778261910780557525 School of Mathematics, Abacws, Cardiff University, CF24 4AG, United Kingdom; 335909682707501476867 Dental Public Health, Faculty of Dentistry, Oral and Craniofacial Sciences, King´s College London, Denmark Hill Campus, Bessemer Rd, London, SE5 9RS, United Kingdom

## Abstract

**Aims** Special care dentistry (SCD) in the United Kingdom (UK) provides care for a range of adults with complex needs, including physically and mentally disabled people, older adults with dementia, adults with medical, emotional, sensory, mental or social impairment, or a combination of these factors, who require care in a range of settings. The aim of this research was to establish a needs-based workforce planning model and tool that focuses on assessing SCD's workforce capacity and capability to deliver tailored dental services for the UK's disabled populace.

**Methods** A predictive analytics tool using Python and HTML was developed to integrate multiple large national datasets to identify key variables for long-term workforce planning and model future SCD demand through 2050. There are currently 13 million disabled adults, 5.9 million of whom are ‘disabled; limited a lot'. National and regional population demand projections for SCD were calculated until 2050 using data mainly from the Office for National Statistics and the 2021–2022 Family Resources Survey. National data on the specialty were obtained from registration and survey data. National and regional resource supply projections for SCD were calculated until at least 2040 recursively from 2018 taking into consideration multiple factors.

**Results** A detailed analysis highlighted several key findings. Under the present scenario, supply aligns with demand only when 5–8% of the ‘disabled; limited a lot' category require complex dental care, suggesting that only a small proportion of needs is currently met by specialists. Planning for the future, for the baseline scenario, where 50% of the ‘disabled; limited a lot' population is considered to have complex needs, even adding 150 specialty trainees per year will not meet projected need until 2040 due to the length of training. If the other half of the ‘disabled; limited a lot' population is assumed to have moderate needs, then upskilling and adding 0.2% of the primary care dentists' pool, or about 80 primary care dentists to the dentists with extended skills (DES) pool per year, would allow this level of moderate need to be met as early as 2034. Strategic workforce redirections, such as even transitioning just 0.1% or 40 primary care dentists into the DES pool per year, could begin to significantly alter the supply-demand dynamics, together with a modest increase in specialty training numbers.

**Conclusions** This study highlights the immense and growing need for SCD and the upskilling of frontline practitioners in caring for adults with additional needs. In essence, while the challenge before us is monumental, it is not insurmountable. Strategic planning, focused training initiatives, and nimble workforce reallocation are pivotal to ensuring that the UK's vulnerable populations receive the specialised dental care they deserve.

**Supplementary Information:**

Zusatzmaterial online: Zu diesem Beitrag sind unter https://doi.org/10.1038/s41415-026-0038-4 für autorisierte Leser zusätzliche Dateien abrufbar.

## Key points

- The population of adults with disabilities is predicted to increase by about 6% by 2050, underscoring the need for integrated planning to meet the growing needs of an ageing and disabled population.
- This modelling-based approach estimates future workforce requirements in special care dentistry, incorporating demographic trends and evolving service demand.
- Developing an appropriate skill mix and service configuration will be critical in addressing workforce gaps and ensuring sustainable care for adults with complex needs.

**Supplementary Information:**

Zusatzmaterial online: Zu diesem Beitrag sind unter https://doi.org/10.1038/s41415-026-0038-4 für autorisierte Leser zusätzliche Dateien abrufbar.

## Background

The United Kingdom's (UK) healthcare system faces a daunting challenge with an escalating shortage of dental professionals,^1^ particularly specialists in special care dentistry (SCD).^2^ This deficit is further exacerbated when viewed against the backdrop of an ageing population,^3^ and particularly for people with disabilities.^4^

### Demographics/ageing

According to mid-2021 population estimates, the population of the UK stands at approximately 67 million, with adults (ages 16 and above) constituting about 80% of this figure, which equates to around 54 million people.^5^ Projections by the Office for National Statistics (ONS) estimate that by 2050, the UK will host a population of around 71.4 million, with adults accounting for approximately 60 million of the population.^2^ Despite the total population witnessing a 6.5% growth over the next three decades, the adult demographic is set to experience an increase of 11.1%, nearly doubling in the same timeframe. More specifically, the population within the 75+ age bracket was recorded at around 5.9 million in 2021, with projections for 2050 at over 10.1 million; nearly double that of the 2021 figure. These statistics, released by the ONS, clearly project a significant surge in the older population within the UK, potentially signalling an upward trend in the number of adults who have additional needs. Furthermore, dementia has been a major and growing public health concern in the UK, with around 900,000 people currently living with the condition.^6^^,^^7^^,^^8^^,^^9^ The number of people diagnosed with dementia is projected to rise to 1.6 million by 2040, particularly afflicting those 65+.^9^ Alarmingly, about one-third of those with dementia remain undiagnosed, denying them timely access to care and support, and suggesting a much higher number of cases than reported.^8^

### Disability

Around one-quarter of the UK population is categorised as disabled.^4^ In this context, disability is defined in line with the Equality Act 2010 as ‘a self-reported physical or mental health condition or illness that has lasted, or is expected to last, at least 12 months and that reduces the ability to carry out day-to-day activities'.^4^ This broad definition includes a range of impairment types, such as mobility, fatigue, dexterity, mental health, memory, hearing, vision, learning, and social/behavioural impairments.^4^ There is a strong correlation between ageing and the onset of impairments later in life.^10^ The rate of disability within the UK population is estimated at 24%, with Scotland and Wales experiencing marginally higher rates at 26%.^4^ While England mirrors the overall UK rate of disability at 24%, a regional breakdown reveals disparities, with the North East and the North West regions experiencing the highest disability rates among the other English regions at 29% and 28%, respectively. In contrast, London reports the lowest disability rate, regionally, at 17%, potentially indicating a younger average population. Conversely, the elevated disability rates in the North East and North West surpassing the national average suggest a higher average age within these regions.

Impairment and disability increase with age.^10^ Caring for people with disabilities represents an important public health issue, given persistent inequalities in access to oral healthcare. As older adults and people with significant disabilities strive to maintain their dentition and overall oral health, the demand for specialised dental care services, particularly SCD, is inevitably on the rise. This shift necessitates a re-evaluation of the current dental care models to ensure they are equipped to meet the evolving needs of the ageing population. In order to address the complex and diverse oral health needs of adults, SCD services must at least attempt to be adaptive, integrating preventive, restorative, and rehabilitative care.^11^

### Workforce supply

Primary care dentistry (PCD) provides general dental care for a majority of the population needing basic dental services. However, it faces inherent limitations in catering to the complex needs of adults with additional needs. PCD often lacks the specialised training, equipment, and resources required to accommodate patients with severe physical or mental impairments.^12^

Thus, informed by a case of need for dental care tailored to individuals with impairments or disabilities, including physical, sensory, intellectual, mental, medical, emotional, or social limitations,^1^ the specialty of SCD was created in the 1990s although formally recognised by the General Dental Council (GDC) in 2008. There are currently 263 specialists on the register of the GDC,^13^ of whom over 75% are female.^12^ At the time of study, there were around 40 specialty trainees in the UK.^12^ Concerns about the serious service gap between need and supply in SCD and the projected growth in this gap, if no major action is taken, have been raised by the specialty.^12^ Given the limited number of SCD specialists, it is imperative to stratify dental care provision.

While dentists with extended skills/special interests in SCD have been proposed, this has not yet been formalised.^14^ Additionally, it has been proposed that there is a role for general dental practitioners to provide the majority of care for people with lower levels of disability.^1^^,^^14^ Primary care dentists, with additional training, will be able to manage more complex cases.

Dentists with extended skills (DES) serve as a bridge between primary care dentists and SCD specialists. They are equipped to handle moderately complex cases, ensuring that individuals with additional needs receive appropriate care without overburdening SCD specialists. Within Community Dental Services (CDS), dental officers and senior dental officers effectively function as DES, delivering care for both adults requiring SCD and paediatric patients,^12^ including those needing general anaesthesia. Strengthening this intermediate workforce – both through formal DES pathways and through support for the existing CDS workforce – would help ensure that individuals with additional needs receive care while allowing specialists to focus on the most severe and multifaceted cases.

The research question is whether the current cohort of SCD specialists, DES, and primary care dentists are, or can be, developed to address this growing demand, both now and in the future. The challenge intensifies as data suggest that the demand for tailored dental interventions is on the rise, primarily driven by an increase in older individuals and the overall rate of disability in the country.

## Methods

Given the increasing ageing population and the limited number of SCD specialists in the UK, it was essential to calculate future need and supply projections in order to address dental workforce capacity planning. Further details of the modelling framework, assumptions, and parameter inputs are provided in the online Supplementary Information. Ethical approval was not required and nor was consent for this modelling study which involved secondary analysis of data in the public domain. The exception was anonymous survey data collected by this research team under ethics committee approval from King's College: ref. MRA-21/22-29397.

### Calculating future need projections

An in-depth needs analysis of dental care services in the UK was conducted, focusing on the SCD demographic. Data from the ONS were used to establish a statistical baseline, identifying the percentage of the population potentially requiring SCD. The UK's total population in 2021 was approximately 67 million, and with an estimated 24% disability rate,^4^ that totals to circa 13 million adults with disabilities. The 2021–2022 Family Resources Survey categorised disability into ‘disabled; limited a little' and ‘disabled; limited a lot' in a ratio of about 55:45, resulting in approximately 7.1 million and 5.9 million adults, respectively.^4^ These categories were further classified into simple, moderate, and complex needs, based on the nature of dental services required, such that primary care dentists would treat those with simple needs, DES would treat patients with moderate needs, and SCD specialists would look after those with complex needs.

Based on published timings by Bannister *et al.*,^15^ alongside input from SCD experts, average times for various dental procedures were calculated, with simple, moderate, and complex procedures taking 20 minutes, 40 minutes, and 60 minutes, respectively. Using these values, alongside the average number of days each type of whole-time equivalent (WTE) dentist typically works per year and the population totals for each need category, we were able to determine the annual workforce requirement for each group. This yielded an estimated need for 1,461 WTE primary care dentists to manage patients with simple needs, 1,291 WTE DES to manage patients with moderate needs, and 2,070 WTE SCD specialists to manage patients with complex needs.

### Calculating future supply projections

The workforce resource pool estimation was regional and temporal in scope, accounting for both present and future years. The latest 2024 GDC data indicated 46,362 registered dentists and 263 SCD specialists available in the UK,^13^ with an estimated 42,000 primary care dentists and 252 active SCD specialists. The number of DES was assumed to be 504, based on expert advice, since there are no data currently available. Supply data for population need until at least 2040 were required to present a snapshot of the shortage, and to assess whether, and when, the need would be met. As data were only available until 2023, the 2018 baseline numbers were recursively projected for all future years. Input parameters, such as the number of dental school graduates, number of dental foundational trainees, and number of SCD trainees, were added to the pool. Output parameters such as retirements, maternity/paternity leave, and joining academia were removed from the supply. The resource pool numbers were adjusted dynamically using probabilistic assumptions by incorporating a Monte-Carlo simulation within the developed tool. The model was parameterised using both GDC data and expert opinion.

### Model implementation

**Fig. 1:**
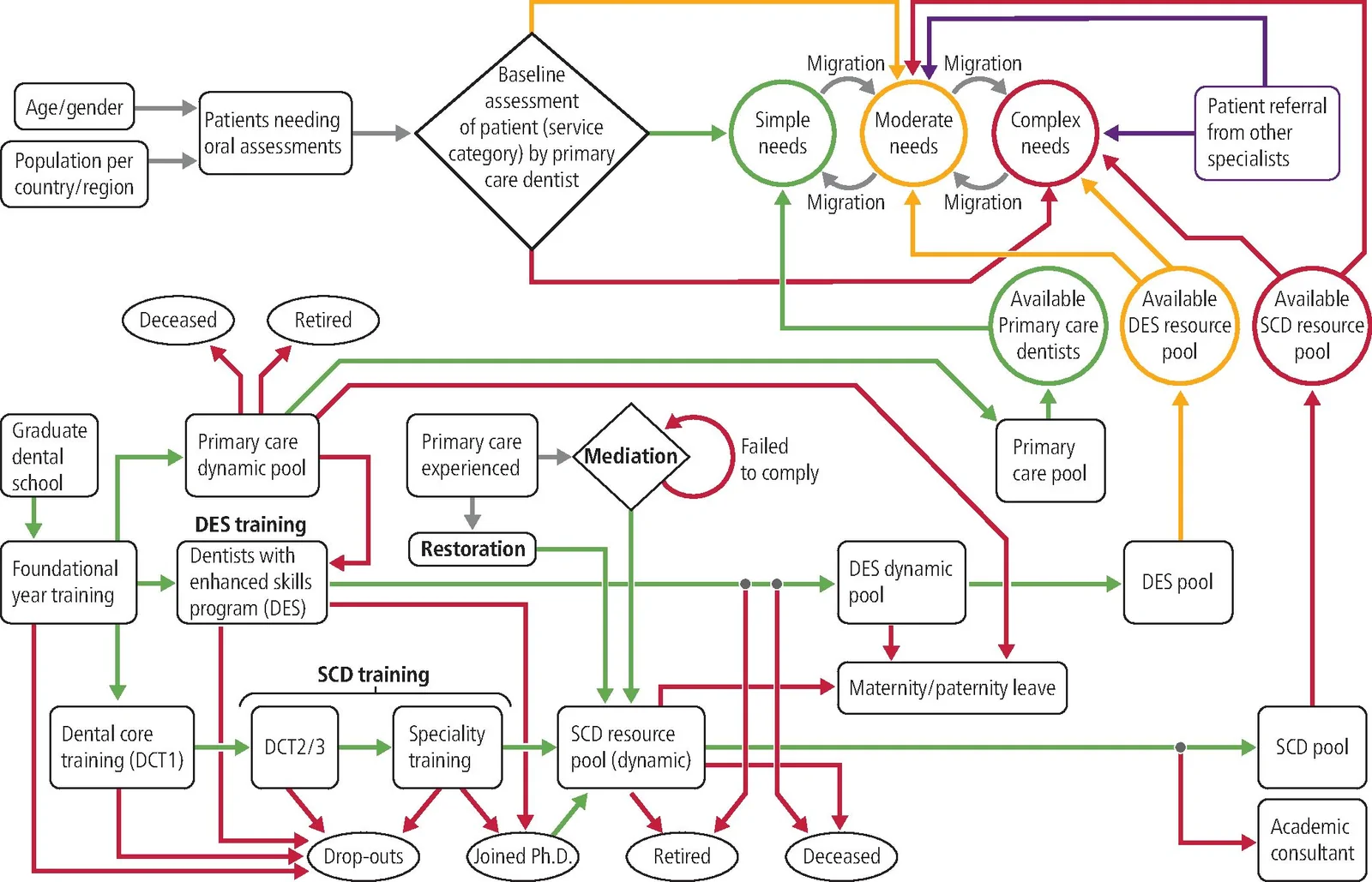
Supply-demand flowchart used in the modelling framework

In order to implement the models mentioned above according to Figure 1, we needed two separate tools that could take various inputs and calculate need and supply for a given year until at least 2040, and needed to be able to run Monte-Carlo simulations to show need and supply for all of the years, ideally as graphs, so users of the tool could plan resources ahead of time.

Since there were many user inputs and graphs to be displayed, HTML forms were used for the main user interface (UI). For the calculations, however, Py-Script was used (Python combined with JavaScript) that can integrate with HTML code.

#### Need model implementation

**Fig. 2:**
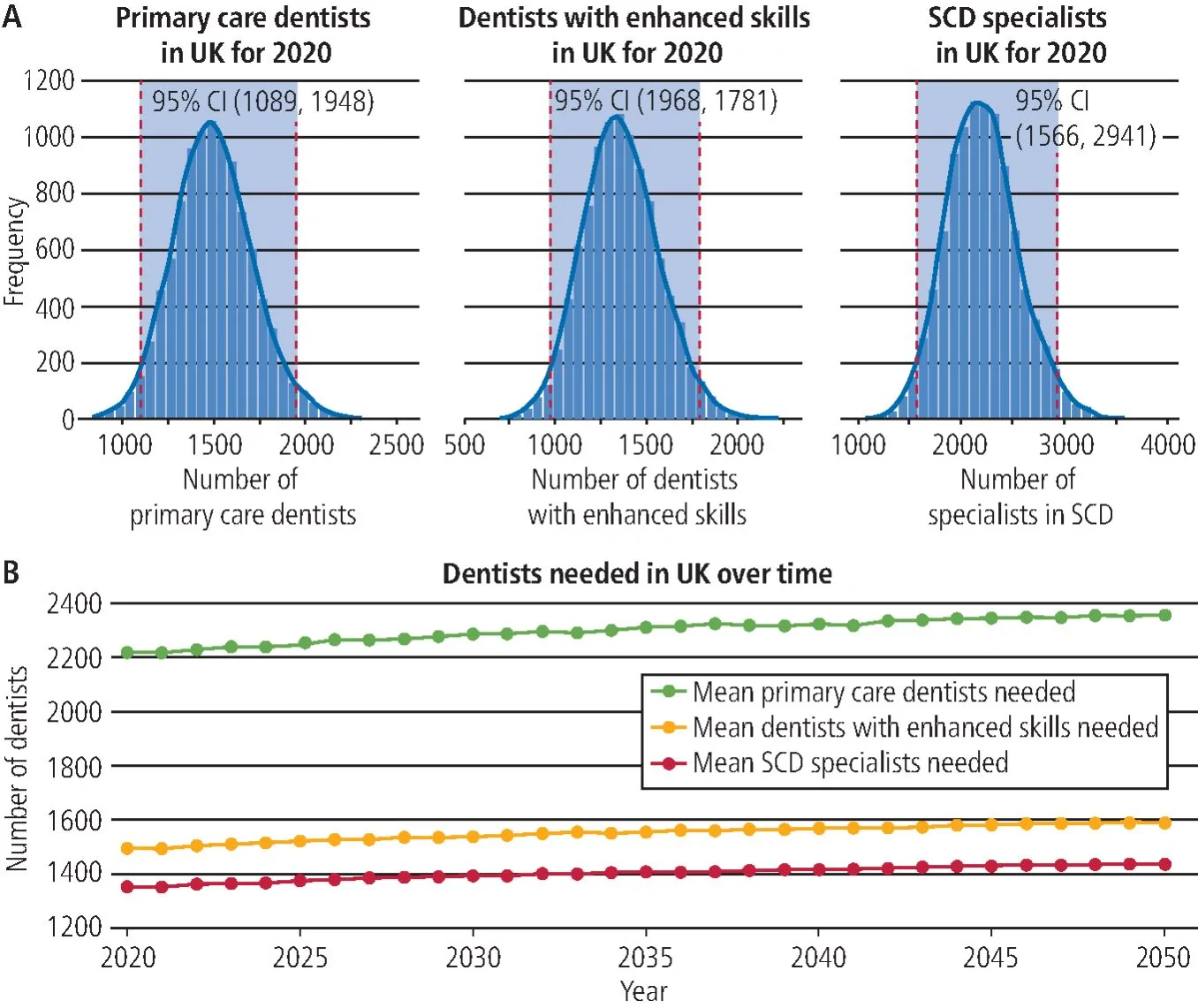
(A, B) Demand tool output – 95% CI graphs and 2020–2050 dentist projection

Upon execution of the demand program, users are presented with a UI to input values, including mandatory fields such as ‘Country/Region' (default: ‘UK'), ‘Year', and for simulations, ‘Start Year for Simulation' and ‘End Year for Simulation'. If mandatory fields are blank, a warning message pops up and the program won't run. Most inputs are prefilled with realistic data for 2020, guiding the user and used for calculations unless changed. The ratio of ‘disabled; limited a lot' population was altered to a 3-way ratio – simple, moderate, and complex categories (e.g., 10:40:50) – only to allow for more flexibility in testing scenarios for the tool. However, for the scenarios presented later, we did not consider any simple cases. Estimates based on user inputs were used to run Monte Carlo simulations to build the 95% confidence interval range around these inputs and fed those values into our computations. The UI for the Need Model has two main parts. The first part computes need for a region for a particular year, displaying the lower and upper bounds, and average for each type of dentist in need as graphs. The second part estimates need pool projections for a range of years and shows two graphs: one showing the average number of each type of dentist needed in the UK over time and another showing box plots for the same with the 95% range (Figure 2).

### Supply model implementation

**Fig. 3:**
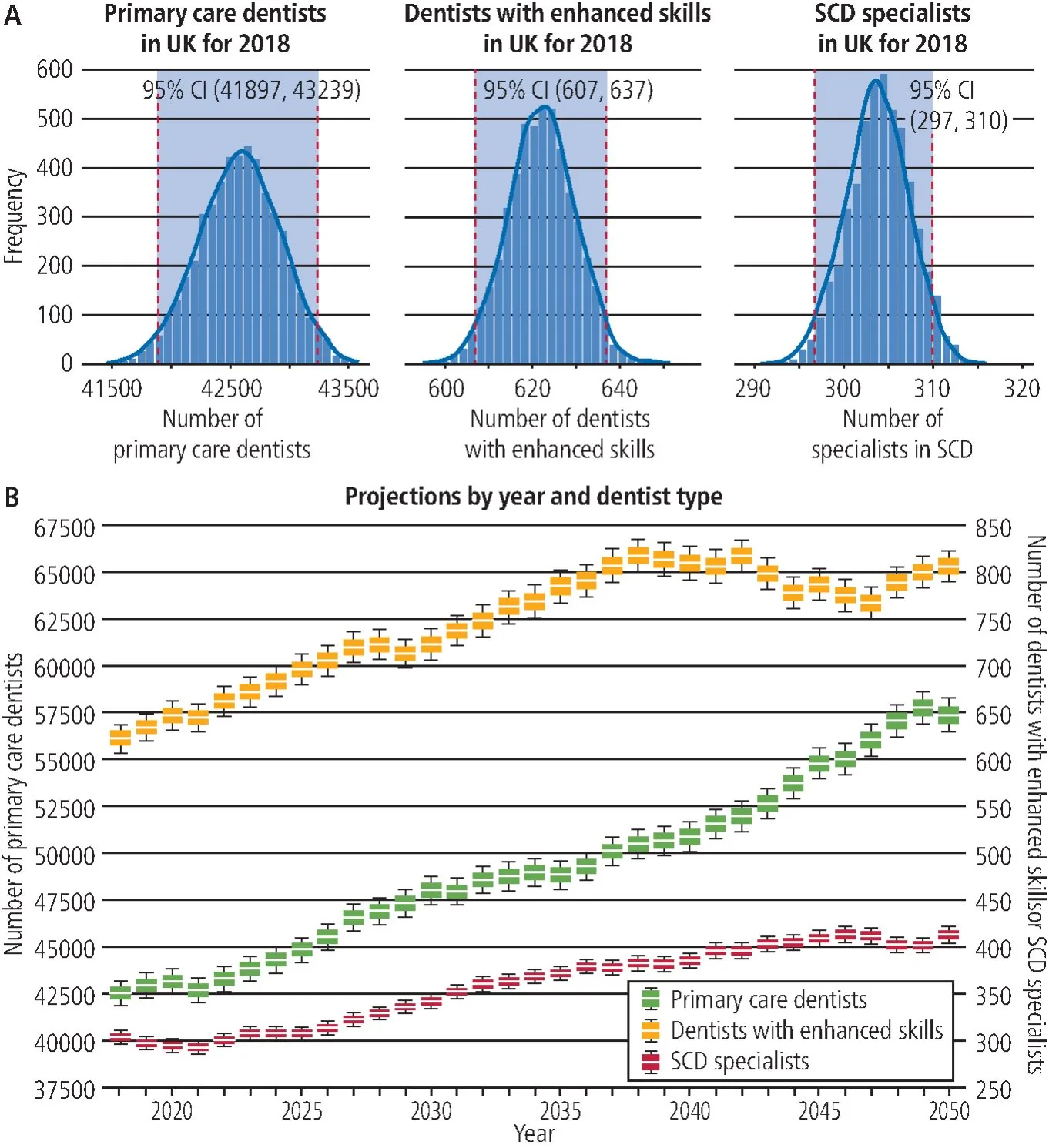
(A, B) Supply tool output – 95% graphs

The Supply Model program, like the Need Model, has two components: 1) estimate the number of each type of dental professionals available at the end of the target year; and 2) simulate supply growth from 2018 to an end year, entered by the user or defaulting to 2040. The program computes supply pool resources recursively, with each year's resources used as input for the next year. For the first part of the program, mandatory fields include ‘Country/Regions' (default ‘UK'), ‘Year', and ‘End Year for Simulation' if running simulations. Like the Need Model, default values were available and based on calculations from 2018 data. Standard deviations were kept at ± 0.5 to simplify calculations for many variables and simulations. Permanent and temporary losses in resource categories were tracked with probabilistic mean and standard deviation inputs. Permanent loss parameters were grouped under ‘Retirement Rate' and temporary ones were grouped under ‘Attrition Rate' in the UI. Additional SCD training posts were added in 2022, and an input field was created for this. Professionals may re-join the workforce each year, adding to the complexity of the supply pool dynamics. Similar to the need model, 95% confidence intervals can be built around the variables by running Monte-Carlo simulations (Figure 3).

## Results

This section details the findings in three parts: model validation, projected need estimates, and scenario-based modelling outcomes.

### Model validation

**Fig. 4:**
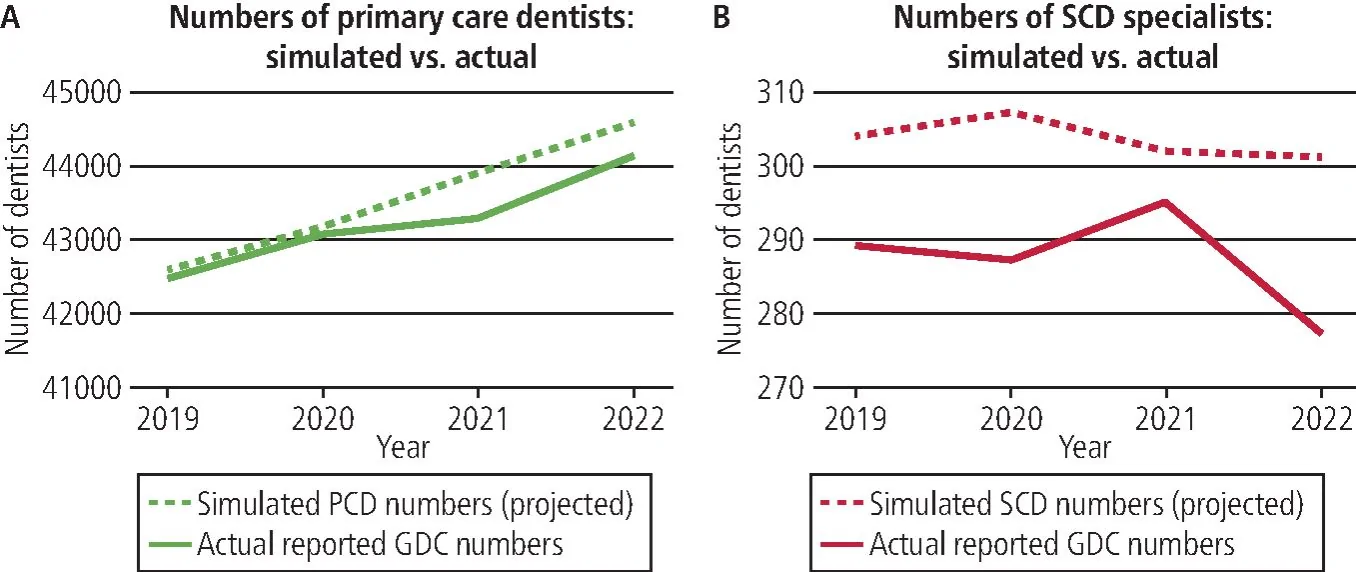
(A, B) Supply tool model validation

The predictive analytics model was validated against GDC-published workforce data for 2019–2022, with the model closely tracking observed trends across all four years. Figure 4 shows the alignment between the simulated and actual values, including the 2022 comparison for primary care dentists (actual: n = 44,125; simulated: n = 44,570) and specialists (actual: n = 277; simulated: n = 301); and this model should, therefore, work as a good foundation to calculate future values.

### Need results – for patients who are ‘disabled; limited a lot' – moderate and complex need

As mentioned earlier, there are currently 7.1 million potential patients who are ‘disabled; limited a little' and 5.9 million patients who are ‘disabled; limited a lot'; these numbers will grow to 7.5 million and 6.2 million, respectively by 2050.

**Fig. 5:**
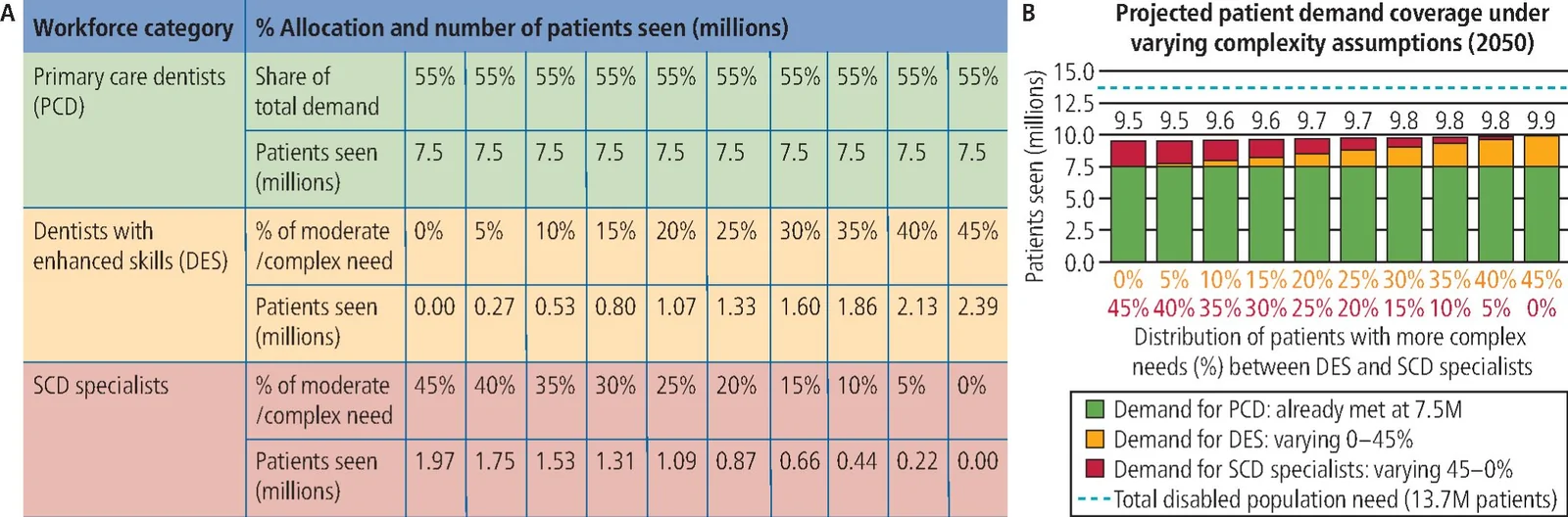
(A, B) Projected patient demand coverage by dental workforce under varying complexity assumptions (2050)

Figure 5 shows that varying the percentage of moderate/complex need (‘disabled; limited a lot') alters the workforce needs such that if 100% of this population had complex needs in 2050, then 6.2 million people would need to be served by specialists.

In the model, all ‘disabled; limited a little' individuals are considered have simple needs and therefore may be seen by routine primary dental care teams. For the ‘disabled; limited a lot' population, the assumption is that these patients would require either moderate or complex care, rather than routine primary care. Various moderate and complex ratios were then tested to examine changes in the estimated need for SCD specialists and DES.

**Fig. 6:**
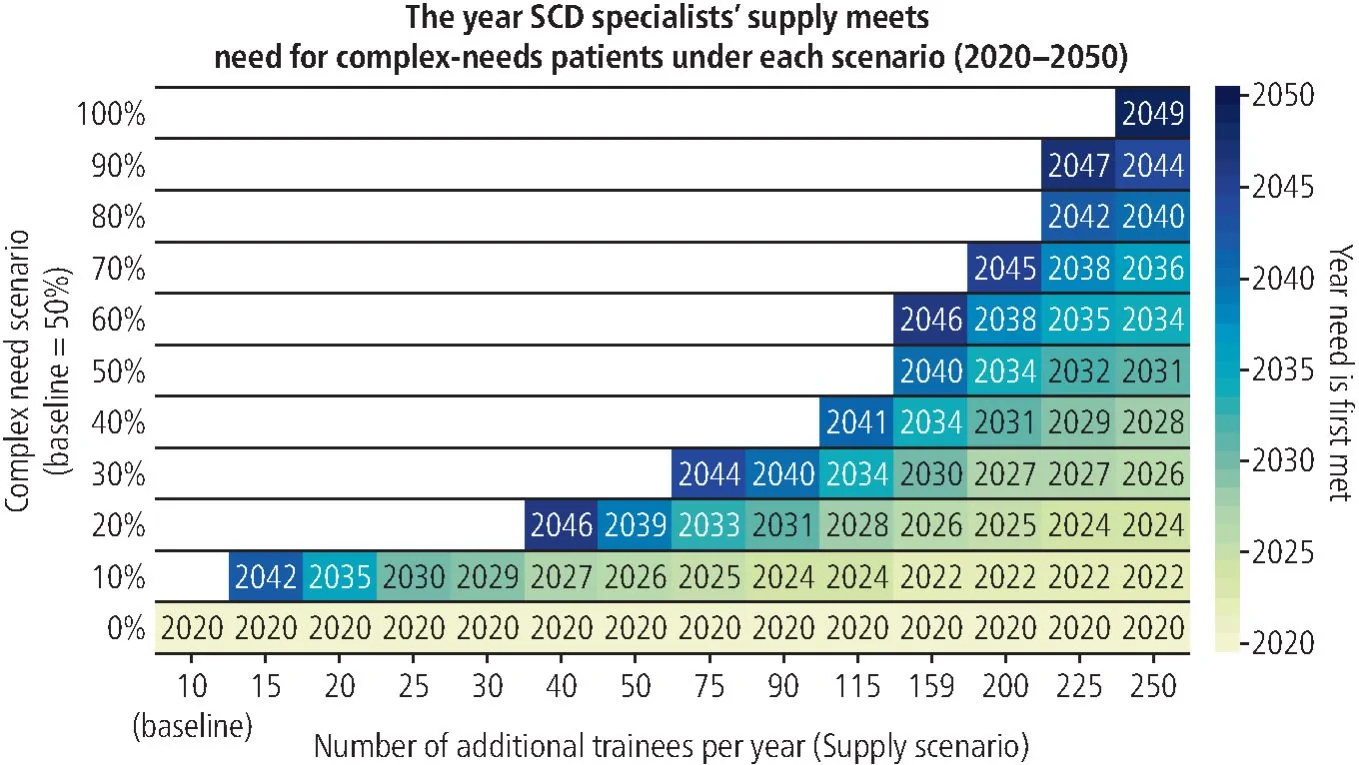
Various projections of number of special care dentistry specialists available to meet complex patients' need by 2050

According to Figure 2, the estimated number of SCD specialists required to meet complex need in 2025 was just above 2,200. As Figure 6 suggests, with current workforce numbers, less than 10% (approximately 5–8%) of the ‘disabled; limited a lot' population with only complex needs is met by the current level of specialists.

**Fig. 7:**
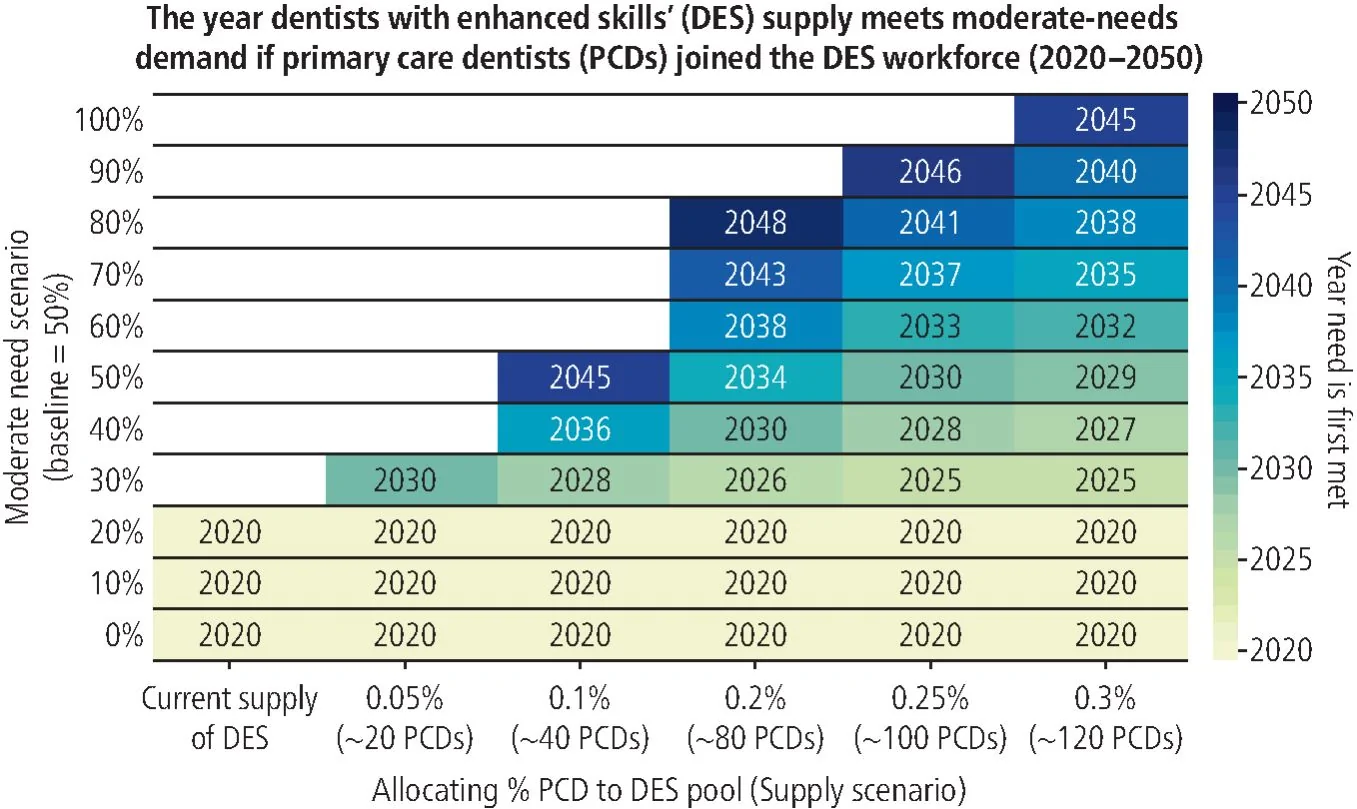
Various projections of number of dentists with extended skills available to meet moderate patients' need by 2050

Figure 7 shows that the current level of DES meets only about 20% of the moderate-care need within the ‘disabled; limited a lot' population. The estimated number of DES required to meet moderate need in 2025 is about 1,400 (Fig. 2).

### Supply results

Considering that there are enough primary care dentists to meet the demand of those who have simple needs, the main focus was on whether there are enough SCD specialists to meet complex need and whether DES can take on a good portion of those patients with moderate need.

### Growing the special care dentistry specialist workforce

Currently, admitting ten trainees per year, resulting in around 40 trainees in the system at any one time, is not sufficient to meet projected need. Thus, increasing numbers of trainees per year was tested to simulate by which year supply meets need for complex needs patients and if demand could be met sooner (Fig. 6).

In the baseline case, where 50% of the ‘disabled; limited a lot' population is assigned to the complex needs category, even with an additional 150 trainees per year, estimated need for care would still not be met until 2040, recognising that current specialists would need to supervise multiple trainees.

Below 150 trainees, this estimated level of need was not being met by 2050.

At one extreme, if 100% of the ‘disabled; limited a lot' population has complex needs, then even with 250 additional trainees per year, need will only be met in 2049. On the other extreme, if only 10% of the ‘disabled; limited a lot' population has complex needs, by adding 15 trainees per year instead of the current ten, this level of need is met in 2042.

### Growing the dentists with extended skills workforce

As mentioned, the estimated number of DES serving around 20% of the ‘disabled; limited a lot' population with moderate needs right now is 504. In order to build the DES workforce, we tested adding small percentages of primary care dentists to the DES pool to see if this need can be met sooner.

For the baseline case where 50% of the ‘disabled; limited a lot' population is assigned to the moderate needs category, adding 0.2% of the PCD pool or about 80 primary care dentists per year to the DES pool can meet need for care as early as 2034, with the DES workforce reaching an estimated 1,463 by that year (Fig. 7).

If 100% of the ‘disabled; limited a lot' population has moderate needs, and we add 0.3% of the PCD pool, or about 120 dentists per year to the DES pool, then it will still take until 2045 for need to be met with 2,889 DES (Fig. 7).

However, if just 40% of the ‘disabled; limited a lot' population has moderate needs, and we add 0.1% of the PCD pool, or about 40 dentists per year to the DES pool, then need is met as early as 2036 with some 1,138 DES (Fig. 7).

As we saw earlier, there is a real shortage of SCD specialists, and if DES can shoulder more of the load in primary care, then the UK would be able to equalise supply and demand much sooner.

## Discussion

### Summary of findings

After testing multiple values for the ‘disabled; limited a lot' population in ‘moderate' and ‘complex' categories, and varying the number of additional SCD trainees joining per year, it is evident that the trainee numbers would need to increase substantially to help manage current and projected need. Currently, the scenario in which supply exceeds or is equal to demand is when only about 5–8% of the ‘disabled; limited a lot' population require specialist care as they potentially have complex needs, which also equates to only around 2.25% of the entire disabled population. While even at our baseline case where 50% of the ‘disabled; limited a lot' population requires specialist care, that equates to 22.5% of the entire population with additional needs, suggesting demand is at least ten times more than what is currently managed. So, it will be necessary to not only increase our resource pool but also emphasise the importance of prevention.

Need is rising. Primary care is potentially less of the issue; although, the recent Adult Oral Health Survey for England 2023, suggests that there is a lot of unmet need in the adult population generally.^16^ Much can be met with ‘generalist' expertise. Moderate and especially complex care are the pressure points/bottleneck. The current SCD specialist workforce is too limited to meet the volume of complex need, under most plausible scenarios. Increasing specialist training is necessary but it will take time to grow this workforce to meet the need. Expanding DES through targeted redistribution from primary care may offer a faster and more practical route for meeting moderate need and thus allow specialists to focus on patients with very complex needs.

### Implications for education and training

A relatively small-scale but strategic redirection of primary care dentists into the DES pool could dramatically shift the supply-demand equilibrium. It would also assist with geographical coverage and enhance dentists' career progression. Migrating just 0.1% of primary care dentists, equivalent to roughly 40 dentists, could either instantly meet the demand or drastically reduce the time required to achieve a balance in selected scenarios. This approach holds immense promise, indicating that even subtle shifts in the workforce allocation could yield significant results. Moreover, facilitating primary care dentists' transition into the SCD realm after a dedicated training period could expedite the resolution of the prevailing shortfall.

The findings underline the need for a dental workforce that is adequately trained and supported to manage an ageing population with increasingly complex needs. It's been emphasised that clinicians require enhanced skills to deliver person-centred care for patients with cognitive or physical impairments.^10^ However, workforce data indicate that the supply of such specialists and extended-skill practitioners remains limited.^13^ And the resourcing of the latter needs to be addressed though appropriate remuneration. This highlights a pressing need to embed special care competencies within undergraduate and postgraduate curricula, supported by continuing professional development and multidisciplinary training and supported by health service policies.

### Implications for nature of care – emphasis on prevention

The shift towards preventive and anticipatory models of care is critical in addressing the oral health needs of people living with disabilities such as dementia and other long-term conditions. The proportion of older adults with complex dependencies is expected to rise substantially.^3^^,^^4^ Thus, this underscores the importance of early intervention, as well as supported self-care, and community-based preventive programmes to reduce reliance on urgent and hospital-based care. National guidance advocates for integrated, prevention-focused approaches – embedding oral health into broader care pathways and dementia strategies.^9^^,^^10^ A greater emphasis on prevention also necessitates collaboration between dental teams, care homes, public health services, and mental health services in order to maintain function and quality of life, rather than solely focusing on episodic treatment.

### Strengths and limitations

This study has a number of strengths, including the use of a transparent modelling framework and probabilistic simulation to estimate future SCD workforce need under different conditions. Separating simple, moderate, and complex need also allows a more clinically meaningful assessment of service demand and workforce requirement. The findings should be interpreted considering several limitations. The model depends on assumptions relating to future disability prevalence, clinician productivity, retirement and attrition, training output, and the extent to which dentists with enhanced skills may absorb part of the projected demand. Furthermore, some inputs were derived from secondary data and expert-informed estimates, particularly where direct workforce data were lacking, most notably DES capacity. While such assumptions were necessary for modelling future scenarios, they introduce uncertainty and mean that the results should be interpreted as indicative planning estimates rather than precise forecasts.

## Conclusion

In conclusion, while the current landscape poses considerable challenges, they are not insurmountable. With focused efforts, the UK can ensure that its vulnerable populations are not deprived of the specialised dental care they critically need. As we chart the course forward, it is imperative to stay rooted in our commitment to delivering unparalleled care and to continuously adapt to the evolving needs of our society.

## Supplementary Information

Supplementary Information (PDF 2MB)

## Data Availability

The data used in this study are derived from publicly available sources listed in the references and online Supplementary Information, together with aggregated input from SCD experts. The survey data which informed the study have already been published and it was determined that the anonymised data that support this article are not available due to ethical considerations that could compromise the identity of research participants.
