# Supplementary material for: Modelling future dental workforce need for adults with special care needs within the UK
Source: Br Dent J. 2026 Nov 13;241(9):630–6. doi: 10.1038/s41415-026-0038-4 (PMC13768167; doi:10.1038/s41415-026-0038-4)
Supplement: Supplementary file 1 [file 41415_2026_38_MOESM1_ESM.pdf]

# Annex

## A1. List of Data Sources

**Annex Table A1. Summary of data sources used in workforce modelling**

| Category | Dataset / Report / Publication | Publisher | Year | Purpose | Reference - URL |
|---|---|---|---|---|---|
| ***Workforce data*** | Registration Statistical Report 2022 | General Dental Council (GDC) | 2023 | Source of registered specialist and DES workforce numbers | GDC (2023): https://www.gdc-uk.org/docs/default-source/annual-reports/gdc_registration-statistical-report-22-23-v3_a.pdf |
| | Registration Statistical Report 2024 | General Dental Council (GDC) | 2024 | Updated workforce growth and trend validation | GDC (2024): https://www.gdc-uk.org/docs/default-source/reports-and-publications/gdc-registration-statistics-report-2024.pdf |
| | SCD Specialist Workforce Survey (Patel et al.) | British Dental Journal | 2025 | Specialist workforce estimates | BDJ(2025): DOI: 10.1038/s41415-025-8723-2. |
| | Dental Public Health Consultant Workforce Review | BASCD | 2023 | Workforce capacity context | BASCD(2023): https://doi.org/10.1922/CDH_00045Gallagher09 |
| | Dental Careers: 2022 Update | Faculty Dental Journal | 2023 | Trends in the dental workforce | Faculty Dental Journal (2023): DOI: 10.1308/rcsfdj.2023.12 |

| | | | | | |
|---|---|---|---|---|---|
| | Distribution of SCD Training Posts | NHS England (WTE) | 2023 | Training pipeline assumptions | National 'Dental Education Reform Programme' (DERP), pp. 1–10. |
| ***Population and demographic data*** | Principal Projection – UK Population in Age Groups | Office for National Statistics (ONS) | 2022 | Projected population by age group to 2050 (used for demand scaling) | ONS (2022): https://www.ons.gov.uk/.../tablea21principalprojectionukpopulationinagegroups |
| | Population Estimates for the UK, England, Wales, Scotland and Northern Ireland: Mid-2021 | Office for National Statistics (ONS) | 2022 | Baseline 2021 population estimates | ONS (2022): https://www.ons.gov.uk/.../mid2021 |
| | Subnational population projections for England | Office for National Statistics (ONS) | 2020 | Demographic modelling for England | ONS(2020) https://www.ons.gov.uk/peoplepopulationandcommunity/populationandmigration/populationprojections/bulletins/subnationalpopulationprojectionsforengland/2018bas |
| | NISRA Census 2021 | Northern Ireland Statistics and Research Agency | 2023 | Disability & health data by age | https://statistics.ukdataservice.ac.uk/dataset/northern-ireland-census-2021-ms-d02-long-term-health-problem-or-disability-by-broad-age-bands |
| | Scotland Census 2011 | National Records of Scotland | 2014 | Population & health | https://www.scotlandscensus.gov.uk/about/2011-census/ |

| | | | | | |
|---|---|---|---|---|---|
| | | | | characteristics | |
| | Family Resources Survey: Financial Year 2021 to 2022 | Department for Work and Pensions (DWP) | 2023 | Disability and complex-need prevalence rates for patient-need estimates | DWP (2023): https://www.gov.uk/government/statistics/family-resources-survey-financial-year-2021-to-2022 |
| ***Oral health and Inequalities*** | Minimising barriers to dental care (Borreani et al.) | BMC Oral Health | 2008 | Access inequalities evidence | BMC Oral Health (2008): http://www.biomedcentral.com/1472-6831/8/7 |
| | Special Care Dentistry: A Professional Challenge | British Dental Journal | 2007 | Background on SCD as a specialty | BDJ (2007): DOI: 10.1038/bdj.2007.426 |
| | Inequalities in dental care provision (Morgan et al.) | Primary Dental Care | 2018 | Context of service distribution | Primary Dental Care (2018): 25(1), 15-21 |
| | Fair Society, Healthy Lives (Marmot Review) | UCL Institute of Health Equity | 2010 | Social determinants framework | https://www.instituteofhealthequity.org/resources-reports/fair-society-healthy-lives-the-marmot-review/fair-society-healthy-lives-full-report-pdf.pdf |
| | The social determinants of oral health (Newton & Bower) | Community Dent Oral Epidemiol | 2005 | Oral health inequalities model | DOI: 10.1111/j.1600-0528.2004.00190.x |
| | Adult Dental Health Survey 2009 | DHSC | 2012 | Historical trends in oral health | DOI: 10.1038/sj.bdj.2012.1088 |

| | | | | | |
|---|---|---|---|---|---|
| ***Condition-specific data*** | Dementia Surveillance Data, England [dataset] | NHS Digital | 2023 | Quantitative prevalence input for special care need estimation | NHS Digital (2023): https://digital.nhs.uk/.../dementia-surveillance-data-2023.csv |
| | Dementia: More Than Just Memory Loss | Older People's Commissioner for Wales | 2016 | Qualitative evidence on barriers and care needs in dementia patients | OPC Wales (2016): https://olderpeoplewales.com/en/publications/dementia-more-than-just-memory-loss.aspx |
| | Briefing – Northern Ireland Budget 2023/24 | Alzheimer's Society | 2023 | Policy and resource context informing future capacity planning | Alzheimer's Society (2023): https://www.alzheimers.org.uk/.../230510-alzheimers-society-briefing-northern-ireland-budget.pdf |
| ***Clinical time and workload parameters*** | Time to Complete Contemporary Dental Procedures – Estimates from a Cross-Sectional Survey of the Dental Team | Bannister C et al., BMC Oral Health | 2023 | Mean procedure-time inputs for modelling clinician capacity and case-mix assumptions | Bannister et al. (2023): https://doi.org/10.1186/s12903-023-03356-3 |
| ***Policy / Guidelines*** | Guides for Commissioning Dental Specialties: Special Care Dentistry | NHS England | 2015 | Defines competency levels and service ratios SCD | NHS England (2015): https://www.england.nhs.uk/wp-content/uploads/2015/09/guid-comms-specl-care-dentstry.pdf |
| | Clinical Standard: Special Care | NHS England | 2022 | Clinical standards | NHS England (2022): |

| | | | | | |
|---|---|---|---|---|---|
| | Dentistry (B1641) | | | for SCD services | https://www.england.nhs.uk/wp-content/uploads/2022/10/B1641-clincial-standard-special-care-dentistry.pdf |
| | Delivering Better Oral Health | Office for Health Improvement & Disparities | 2021 | Prevention toolkit baseline | https://doi.org/10.1038/s41407-021-0751-4 |
| | Specialties: Relevance and Reform in the UK | Faculty Dental Journal | 2017 | Policy relevance of specialties | https://doi.org/10.1308/rcsfdj.2017.10 |
| | BSDH Guidelines for Special Needs Oral Health | BSDH | 2018 | Clinical guidance for SCD services | |
| | Improving Dental Care & Oral Health – Call to Action | NHS England | 2014 | Workforce & prevention strategy | https://www.england.nhs.uk/wp-content/uploads/2014/02/imp-dent-care.pdf |

## A2. List of Assumptions

**Annex Table A2. Key modelling assumptions used in workforce and demand projections**

| No. | Assumption | Rationale / Basis |
|---|---|---|
| 1 | Rate of disability and complex need in elderly populations assumed constant (no major increase beyond demographic ageing) | While evidence suggests prevalence of disability rises with age, improvements in preventive oral health and general health outcomes are expected to mitigate functional decline. This balances risk growth with anticipated gains in preventive and community care. |

| | | |
|---|---|---|
| 2 | Productivity per clinician (measured as patients treated per year) remains at current mean estimates | Procedure-time and workload estimates (Bannister et al., 2023) are assumed representative, as technological improvements (e.g., digital records, sedation advances) roughly offset rising case complexity. |
| 3 | The number of Dentists with Extended Skills (DES) currently available is estimated to be double the number of specialists available | There is no published workforce data on Dentists with Extended Skills. With expert input, it is assumed to be double that of the number of SCD specialists. |
| 4 | The proportion of dentists with Enhanced Skills (DES) scales gradually with demand up to 45% complexity share | Models flexibility in skill-mix adaptation. Reflects NHS emphasis on *upskilling existing workforce* rather than full specialist substitution. |
| 5 | Primary Care Dentists (PCDs) maintain a fixed 55% share of total demand coverage | Reflects stable capacity for routine care, assuming preventive interventions and recall scheduling efficiency remain consistent with Delivering Better Oral Health guidance (OHID, 2021). |
| 6 | Demand for SCD Specialists decreases proportionally as DES workforce expands | Reflects strategic redistribution of moderate-complexity care to mid-level providers. |
| 7 | Total population with moderate/complex need grows proportionally to national demographic projections (ONS, 2022) | Based on principal UK population projections by age group; assumes no unexpected public health shocks or pandemics significantly altering disability burden. |
| 8 | No major policy disruptions (e.g., NHS contract restructuring, Brexit-like migration shocks) altering dentist supply | Conservative baseline assumption; allows model focus on service complexity and demand, not workforce shocks. |
| 9 | Training output for DES and SCD remains proportional to current trends | Although NHS England is expanding discussions around dental workforce reform, published training post numbers and recent DERP updates show relatively slow year-on-year growth. Assuming proportional continuation (rather than rapid expansion) avoids overestimating future specialist/DES supply in the absence of confirmed policy changes. |

| | | |
|---|---|---|
| 10 | No major change in the proportion of dentists working part-time vs. full-time | Recent GDC workforce reports and Ashtari et al. (2023) show increasing flexibility and portfolio careers, but the overall full-time/part-time ratio has remained broadly stable over the past decade. Maintaining this ratio prevents artificially boosting capacity based on optimistic assumptions about full-time equivalents (FTE). |
| 11 | Prevalence of multimorbidity assumed to increase modestly but not enough to drastically alter service utilisation rates | ONS and Marmot Review data suggest rising multimorbidity, especially in deprived areas, but improved chronic disease management and preventive frameworks (e.g., Delivering Better Oral Health toolkit) may offset the impact on dental service need. A modest increase reflects realistic clinical trends without overestimating the burden of complexity. |

## A3. Full Methods

### Methodology:

Given the increasing ageing population and diminishing numbers of SCD specialists in the UK, it was essential to calculate future need and supply projections in order to address dental workforce capacity planning.

1. **Calculating Future Need Projections:**

The first step was an in-depth need analysis of dental care services in the UK, focusing on a specific demographic that needs services from Special Care Dentistry. The analysis spanned multiple years, starting from the current year, and extending into future projections. Data for these assessments had been sourced from the ONS and covered regional variations as well. A statistical baseline identifying the percentage of the population requiring SCD was established. The population was segmented into age brackets – 0-15, 16+, and 75+, with the focus on the adult and elderly populations. As mentioned earlier, UK's total population in 2021 was about 67 million, which included about 54 million adults. The rate of disability in the UK is estimated to be 24%, which totals to around 13 million adults with disabilities.

The 2021-2022 Family Resources Survey had categorized disability into two types: 'Disabled; limited a little' and 'Disabled; limited a lot' in a ratio of about 55:45. Classifying the 13 million adults with disabilities in the UK into these two categories with this ratio gave us around 7.1 million adults considered 'Disabled; limited a little' and about 5.9 million considered 'Disabled; limited a lot'. These two categories were further classified into three different categories based on the nature of dental services they might need: simple, moderate, and complex.

• **Simple Needs**: The 7.1 million patients considered 'Disabled; limited a little' were bracketed into this category assuming their dental needs would be simpler in nature, with procedures such as basic examinations, assessments, and routine cleanings, that can be effectively managed by primary care dentists.

• **Moderate Needs**: A portion of the 5.9 million 'Disabled; limited a lot' population would consist of those needing services more involved than Simple Needs category but moderately demanding like Restorative Work, Root Canal Treatment, Periodontal Treatment, Partial Dentures, and Basic Crowns. These procedures necessitate specialized skills not typically found amongst primary care dentists and are thus best suited for professionals categorized as dentists with extended skills.

• **Complex Needs**: The remainder of the 5.9 million 'Disabled; limited a lot' population are those who are severely disabled and therefore have complex needs. Their dental needs would be extremely intricate and time-consuming. This third category addresses advanced procedures that demand specialized training and equipment, often requiring multiple appointments for completion. Dental professionals specialized in SCD are probably best equipped to manage these complex cases, particularly when patients have severe disabilities, significant co-morbidities, or challenging behavioural conditions.

Note that it is possible for patients classified under one category to migrate to another after the initial assessment by the dentist or if conditions change.

"Time to complete contemporary dental procedures – estimates from a cross-sectional survey of the dental team" gave a very detailed insight into the average times of various types of common dental procedures when completed on adults without disabilities, with the average times increasing as the complexity of the dental procedures increased. To calculate how many patients a whole time equivalent (WTE) dentist working 8 hours per day can see in a single day, it was necessary to know the average time spent in one appointment for each of the three categories of patients. The different dental procedures were split into three groups as well based on the complexity level of the services rendered: simple, moderate, and complex.

The average times for each of these groups were calculated and assigned accordingly to the type of patient and type of dentist. From this, it was possible to calculate the number of patients seen in a year and then that combined total with the special needs' population

numbers, we were able to determine the demand for each type of dentist. The timing for each procedure category is listed below:

- Simple Need Procedure: 5.75 mins
- Moderate Need Procedure: 14.1 mins
- Complex Need Procedure: 59.86 mins

However, if these specific average timings were used, then it was evident that the number of patients a dentist can see per day for simple and moderate need cases were extremely unrealistic with 86 patients/day for a WTE primary care dentist working non-stop and only tending to patients with simple needs and 34 patients/day for a dentist with extended skills only tending to those with moderate needs. These figures were unnaturally high because they don't take disability into consideration as Bannister's study was done on adults without disabilities and it was initially assumed that a simple need patient would only require simple procedures. However, a typical dental appointment would at the very least comprise of some simple and some moderate procedures even for a simple need patient. More involved appointments might entail a mix all procedure types. All of these would lend to higher average times, in line with a typical appointment time slot at the dentists. So, for a more realistic scenario, as informed by Bannister's paper and specialist SCD experts, the following values were used:

- Simple Need Procedure: 20 mins
- Moderate Need Procedure: 40 mins
- Complex Need Procedure: 60 mins

From the timings above, the number of patients seen per day by a primary care dentist, dentist with extended skills, and a SCD specialist was 24, 12, and 8, respectively. The number of weekdays in a year is 262. However, factoring in days where dentists don't actively look at patients, the number of days in a year that a primary care dentist, dentist with extended skills, and a SCD specialist works would be 202, 187, and 175, respectively. Multiplying these three numbers with the number of patients seen per day resulted in the number of patients seen per year. A WTE primary care dentist can see 4848 patients with simple needs per year. Similarly, a WTE dentist with extended skills can see 2244 patients with moderate needs per year, and a WTE SCD specialist can see 1400 patients with complex needs.

Finally, to calculate need per year, the adult simple needs' population was divided by the number of patients a WTE primary care dentist sees, which gave us 1461, and after repeating with the other figures, we had 1291 patients with moderate needs for a dentist with extended skills, and 2070 patients with complex needs for a SCD specialist.

**2. Calculating Future Supply Projections:**

The workforce resource pool or supply estimation was regional and temporal in scope, accounting for both present and future years. However, any projections had to be done recursively as there was data available by the General Dental Council (GDC) from only 2018 to 2023 so far.

Latest 2024 GDC data suggested that there are currently 46,362 registered dentists and 263 SCD specialists available in the UK. Note that the number of primary care dentists is slightly inflated as it includes about 10% of those who are specialists. So, the actual value should be around 42,000 primary care dentists. The number of SCD specialists is also slightly higher than the actual value as the GDC's report include those who have retired or are no longer practicing. The actual number of active specialists in the UK is 252, which was estimated by SCD experts. Currently, there are no official dentists with extended skills' positions – some dentists just volunteer some of their time. As there is no information on the number of dentists with extended skills available currently, we assumed that it was double the number of SCD specialists available or 504 for now.

Considering, we had data for population need until at least 2040 for all countries and regions, we also needed supply data for the same to present a snapshot of the shortage throughout the years and to find out when and if need is being met and if not, under what scenarios would need be met. Since supply pool projections data until 2040 or later was not available, we took 2018 data as the baseline and recursively computed projections for all future years. The 2018 baseline numbers for primary care dentists, dentists with extended skills, and SCD specialists in the UK were dynamically adjusted with the impact of certain parameters (shown below) that resulted in the increment or decrement of the count in these pools and this gave us estimates for 2019. And then we used 2019 data to calculate 2020 data and so forth.

The following parameters were considered where "input parameters" get dynamically added to the pool and "output parameters" get dynamically remove from the pool.

**Input Parameters (add to pool)**:

a. Dental school graduates
b. Foundational Year Training (DFT)
c. Dental Core Training 1 -> Dental Core Training 2/3 -> Specialty training
d. Restoration and Mediation
e. Any re-joiners

**Output Parameters (remove from pool)**:

a. Trainee Dropouts (assuming 0)
b. Retired / Not practicing
c. Maternity / Paternity leave
d. Sabbaticals
e. Joining PhD / academia
f. Deceased
g. Failed to comply
h. Becoming academic consultant

Variables that shifted the resource pool numbers were mostly general ones that were not specific to the type of dentist and those included retirement, maternity/paternity leave, sabbaticals, going into academia, death, restoration, and mediation. Some of these inputs were temporary as there was a high chance of most of the dentists joining the workforce again. These dynamics were modelled using probabilistic assumptions based on average known data from GDC, etc.

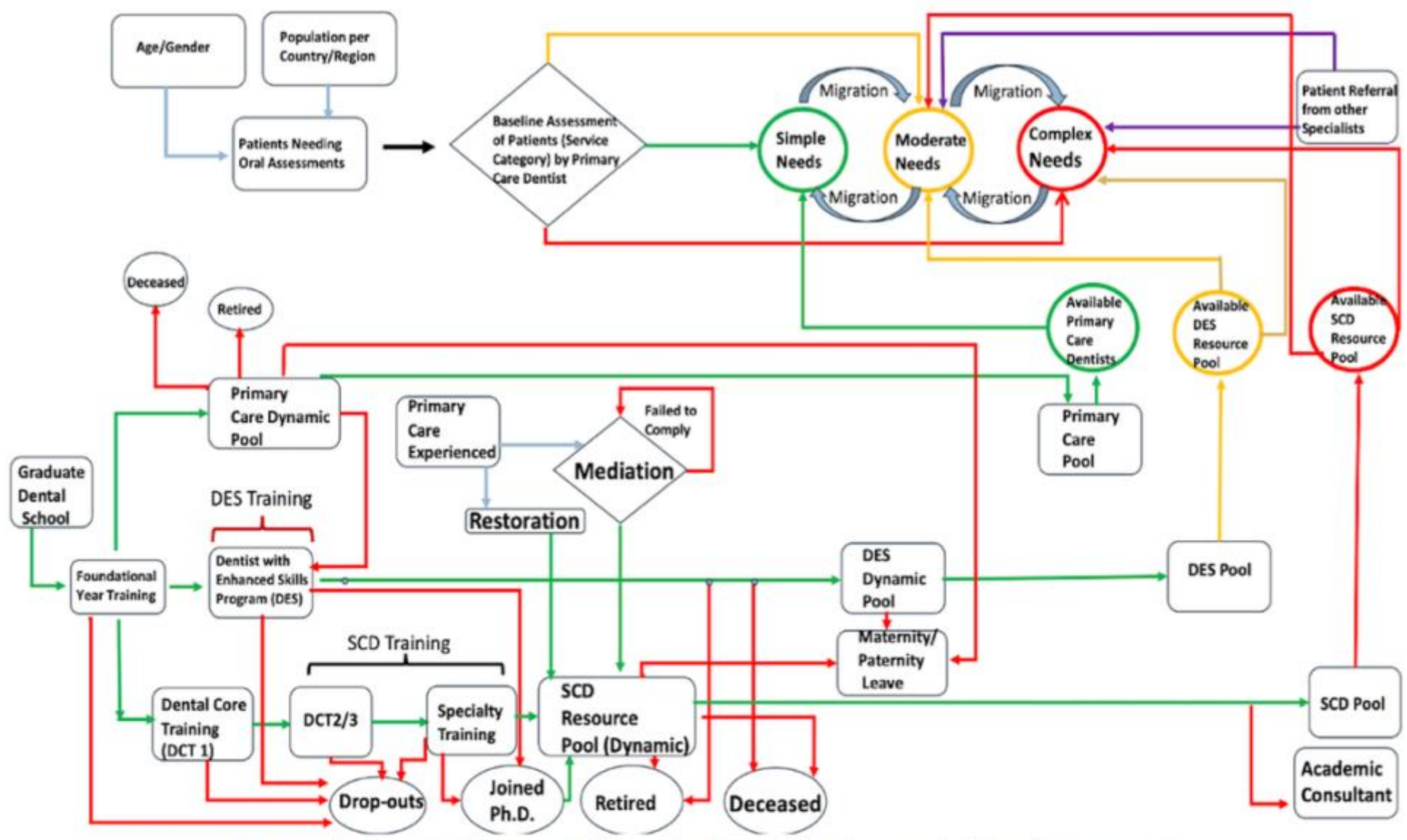


*Figure 1 Supply-Demand Flowchart used in the modelling framework*

Description: A demand/supply flowchart showing how different patients are classified and which factors are contributing to dentists' resource pools. Figure created by the authors for this manuscript. The broader modelling approach was informed by Gallagher et al. (2023).
Data Sources: ONS, GDC, Gallagher et al., etc.
Note: Most of the red lines in the supply portion or bottom mean numbers are being removed from the resource pools and green means numbers being added to the pool. Red also represents SCD resource pool, yellow for the dentists with extended skills resource pool, and green for primary care dentists' resource pool. Only in the top half or demand half, red represents those with complex needs, yellow for those with moderate needs, and green for those with simple needs.

## 3. Model Implementation:

In order to implement the models mentioned above according to Fig. 1, we needed two separate tools that can take various inputs and calculate need and supply for a given year until at least 2040 and we needed to be able to run simulations to show need and supply for all of the years, ideally as graphs, so users of the tool could plan resources ahead of time.

Since there were many user inputs and graphs to be displayed, HTML forms were utilized for the main user interface. For the calculations, however, Py-Script was used, which is basically Python combined with JavaScript that can integrate with HTML code.

### i) Need Model Implementation:

Upon execution of the demand program, users are presented with a user interface or UI to input values. Mandatory fields include 'Country/Region' (default: 'UK'), 'Year', and for simulations, 'Start Year for Simulation' and 'End Year for Simulation'. If mandatory fields are blank, a warning message pops up and the program won't run. Most inputs are prefilled with realistic data for 2020, guiding the user and used for calculations unless changed. The ratio of 'Disabled; limited a lot' Population was altered to a 3-way ratio – 'Ratio of 'Disabled; limited a lot' Population in Simple, Moderate, and Complex categories (ex. 10:40:50)' only for the tool (Fig. 2) for more flexibility in testing scenarios. However, for the scenarios presented later, we did not consider any simple cases.

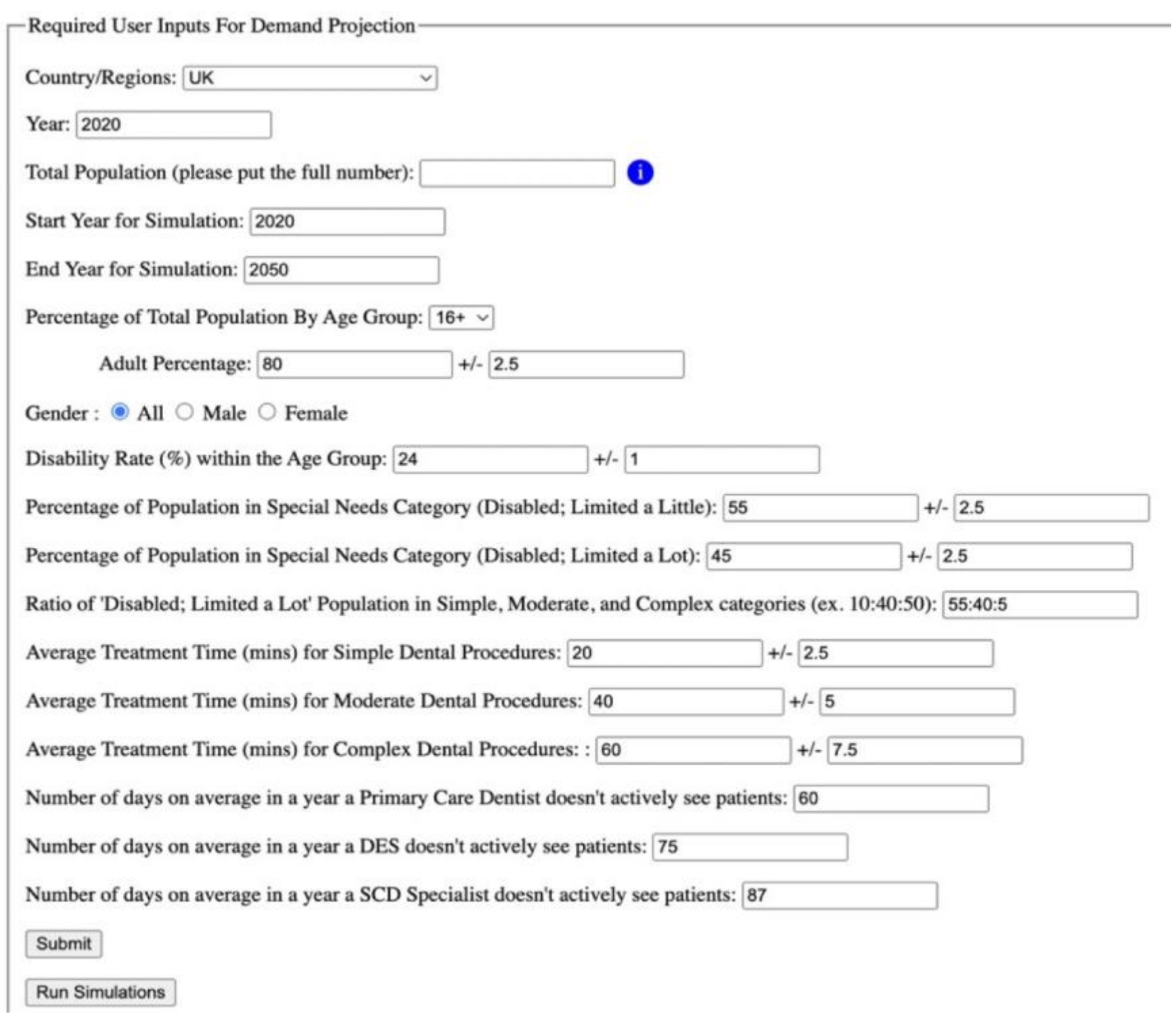


*Figure 1 Demand Tool UI*

Description: UI for the Demand or Need Tool – takes in relevant data and projects population need for that year and/or all years from 2020-2050
Data Sources: ONS, Family Resources Survey, Bannister's Paper, SCD Experts
Note:

Also, since many of these user inputs were based on estimates, we added a small margin of variation and ran Monte Carlo simulations (10,000 runs in Py-Script using normal distribution) to build the 95% confidence interval range around these inputs and fed those values into our computations.

The UI for the Need Model has two main parts. The first part computes need for a region for a particular year. As shown in Figures 3 and 4, the lower and upper bounds, and average for each type of dentist in need are calculated and displayed as graphs.

Output:

Region selected: UK

Year selected: 2020

Total Population of UK in 2020: 67081234

Total population by age group: 53664987

Selected Gender: All

Total Disabled Population based on 24% Rate: 12879596

Population that is Disabled; limited a little: 7083777

Population that is Disabled; limited a lot: 5795818

Ratio of Disabled a little to Moderately Disabled to Severely Disabled: 0:50:50

Average time spent on simple dental procedures: 20

Average time spent on moderate dental procedures: 40

Average time spent on complex dental procedures: 60

Number of patients with simple needs seen per day: 24

Number of patients with moderate needs seen per day: 12

Number of patients with complex needs seen per day: 8

95% CI for Primary Care Dentists required: (1089, 1948). Mean is 1492

95% CI for Dentists with Enhanced Skills required: (968, 1781). Mean is 1351

95% CI for SCD Specialists required: (1566, 2941). Mean is 2209

*Figure 2 Demand Tool Output*

Description: Output of Demand/Need Tool shows the 95% confidence interval range and average number of each type of dentist required to satisfy need
Data Sources: ONS, Family Resources Survey, Bannister's paper, SCD Experts
Note:

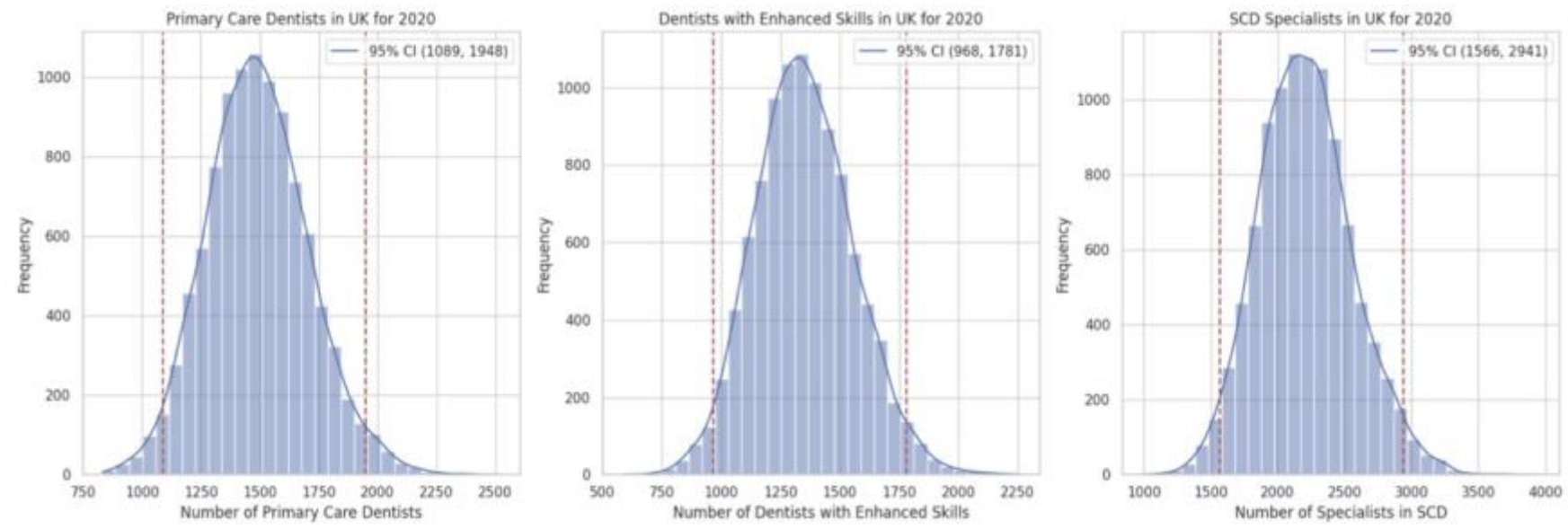


*Figure 3 Demand Tool Output - 95% CI Graphs*

Description: This is also part of the demand tool output and it shows the 95% confidence interval for each of the 3 types of dentists
Data Sources: ONS, etc.
Note:

The second part, once "Run Simulations" is clicked, estimates need pool projections for a range of years starting from the "Start Year for Simulation" and ending at "End Year for Simulation, as provided by the user, and shows two graphs: one showing the average number of each type of dentist needed in the UK over time and another showing box plots for the same with the 95% range.

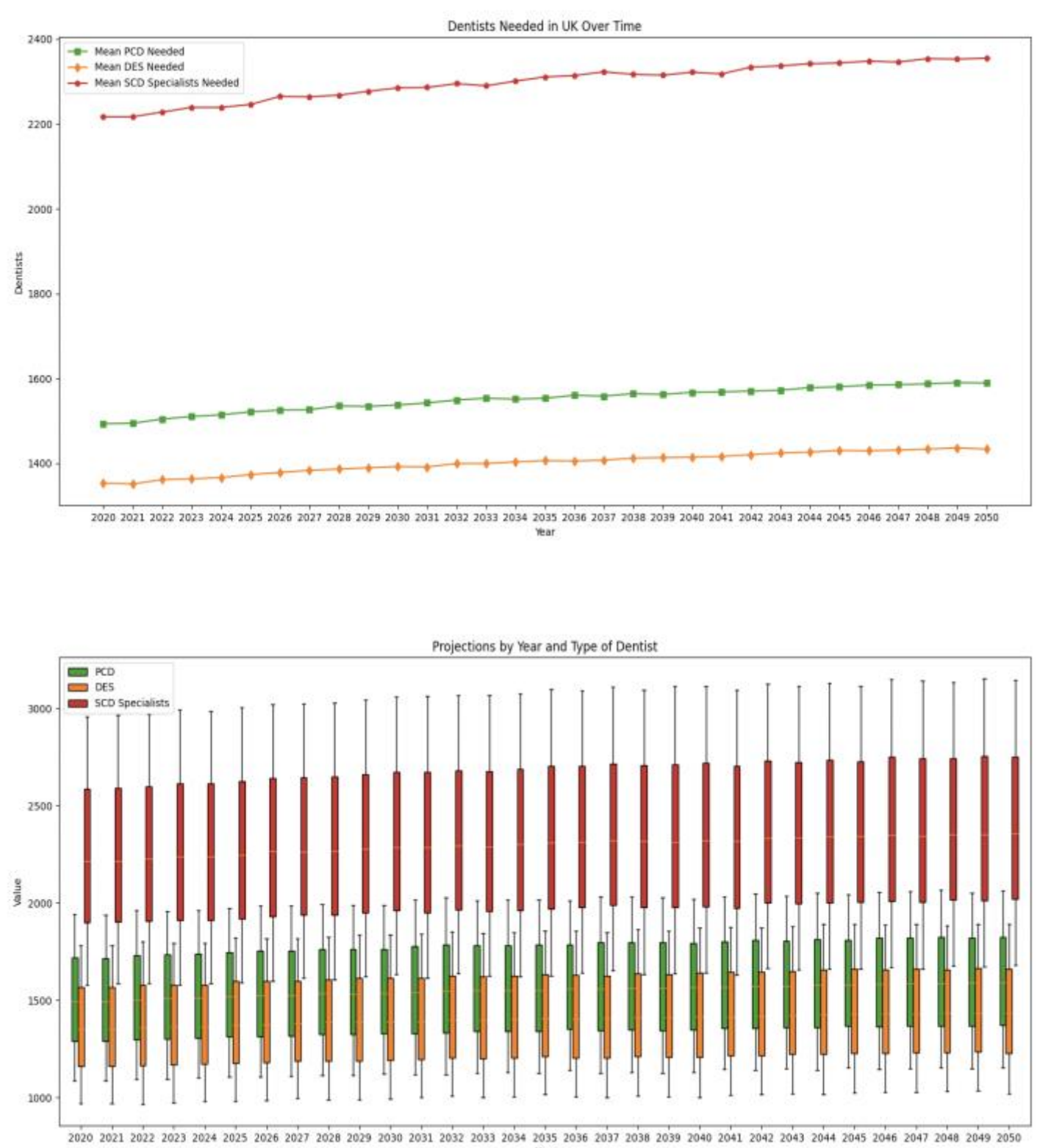


*Figure 4 Number of All types of Dentists needed Projections 2020-2050*

Description: Top graph (5a) show the average number of primary care dentists, dentists with extended skills, and specialists required to meet need in the UK from 2020-2050. Bottom graph (5b) is the same but just shows the 95% CI range of each type of dentist for meeting need from 2020-2050
Data Sources: ONS, Family Resources Survey
Note:

### ii) Supply Model Implementation:

The Supply Model program, like the Need Model, has two components: (1) Estimate the number of each type of dental professionals available at the end of the target year and (2) Simulate supply growth from 2018 to an end year, entered by the user or defaulting to 2040. The program computes supply pool resources recursively, with each year's resources used as input for the next year.

For the first part of the program, mandatory fields include 'Country/Regions' (default 'UK'), 'Year', and 'End Year for Simulation' if running simulations. Like the Need Model, default values were available and based on calculations from 2018 data. Standard deviations were kept at +/- 0.5 to simplify calculations for many variables and simulations. Permanent and temporary losses in resource categories were tracked with probabilistic mean and standard deviation inputs. Permanent loss parameters were grouped under "Retirement Rate" and temporary ones were grouped under "Attrition Rate" in the UI (Fig. 6).

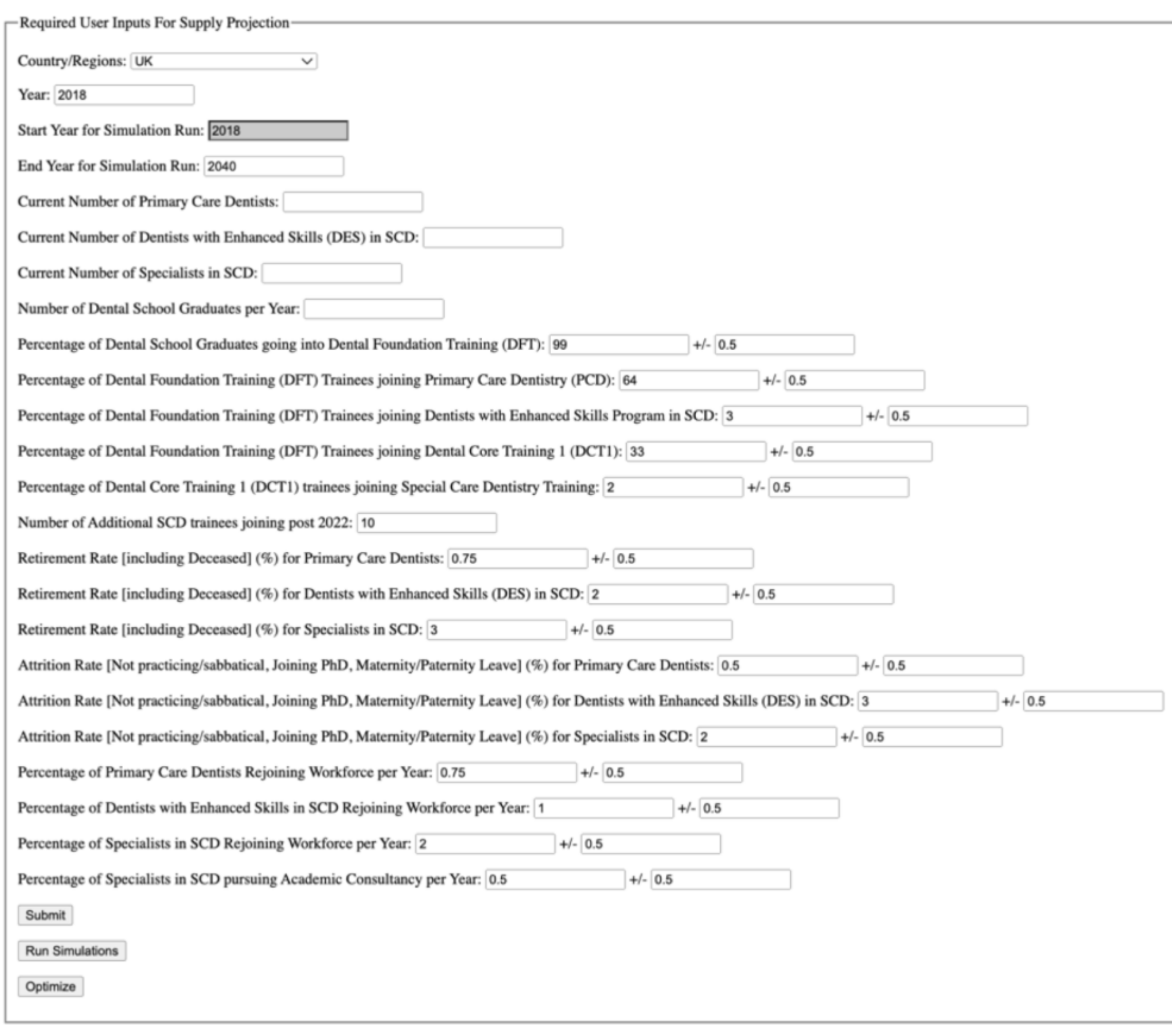


*Figure 5 Supply Tool UI*

Description: UI for the supply tool – calculating the available resource pools for each type of dentist: primary care dentists, dentists with extended skills, and specialists
Data Sources: ONS, GDC, expert input
Note:

Additional SCD training posts were added in 2022, and an input field was created for this. Professionals may re-join the workforce each year, adding to the complexity of the supply pool dynamics, which could best be modelled using a simulation program. Similar to the need model, 95% confidence intervals can be built around the variables by running Monte Carlo simulations.

Output:

Region selected: UK

Year selected: 2018

Current Number of Primary Care Dentists in 2018 in UK: 42119

Current Number of DES in SCD in 2018 in UK: 614

Current Number of Specialists in SCD in 2018 in UK: 307

Number of Dental School Graduates per Year: 1100

Number of DFT Trainees per Year: 1089

Number of DFT Trainees joining PCD per Year: 696

Number of DFT Trainees joining DES Program in SCD per Year: 32

Number of DFT Trainees joining DCT1 Training per Year: 359

Number of DCT1 Trainees joining SCD Training per Year: 7

Number of Primary Care Dentists retiring per Year: 315

Number of DES in SCD retiring per Year: 12

Number of SCD Specialists retiring per Year: 9

Number of Primary Care Dentists attritions per Year: 210

Number of DES in SCD attritions per Year: 18

Number of SCD Specialists attritions per Year: 6

Number of Primary Care Dentists rejoiners per Year: 315

Number of DES in SCD rejoiners per Year: 6

Number of SCD Specialists rejoiners per Year: 6

Number of SCD Specialists becoming Academic Consultants: 1

95% CI for Primary Care Dentists available: (41897, 43239). Mean is 42585.

95% CI for Dentists with Enhanced Skills available: (607, 637). Mean is 623.

95% CI for SCD Specialists available: (297, 310). Mean is 304.

*Figure 6 Supply Tool Output*

Description: Supply tool output shows the 95% CI and average number of PCDs, DES', and Specialists available
Data Sources: GDC, ONS
Note:

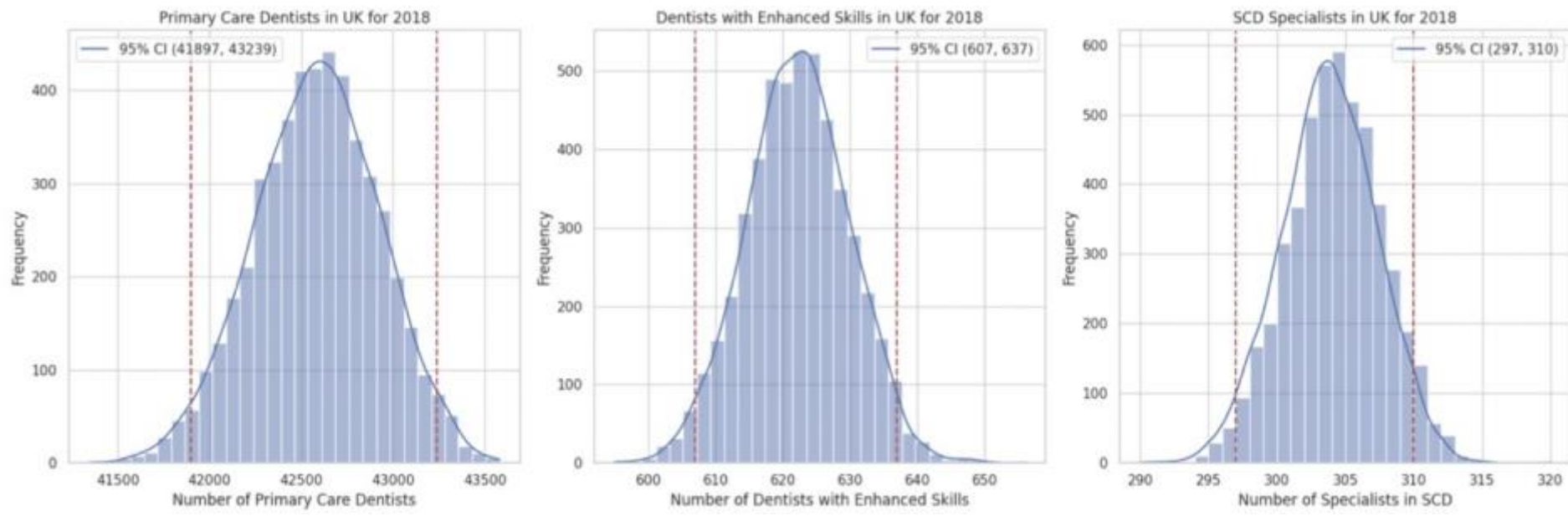


*Figure 7 Supply Tool Output – 95% Graphs*

Description: This is also part of the supply tool output - graphs showing the 95% CI for each type of dentist
Data Sources: ONS, GDC
Note:

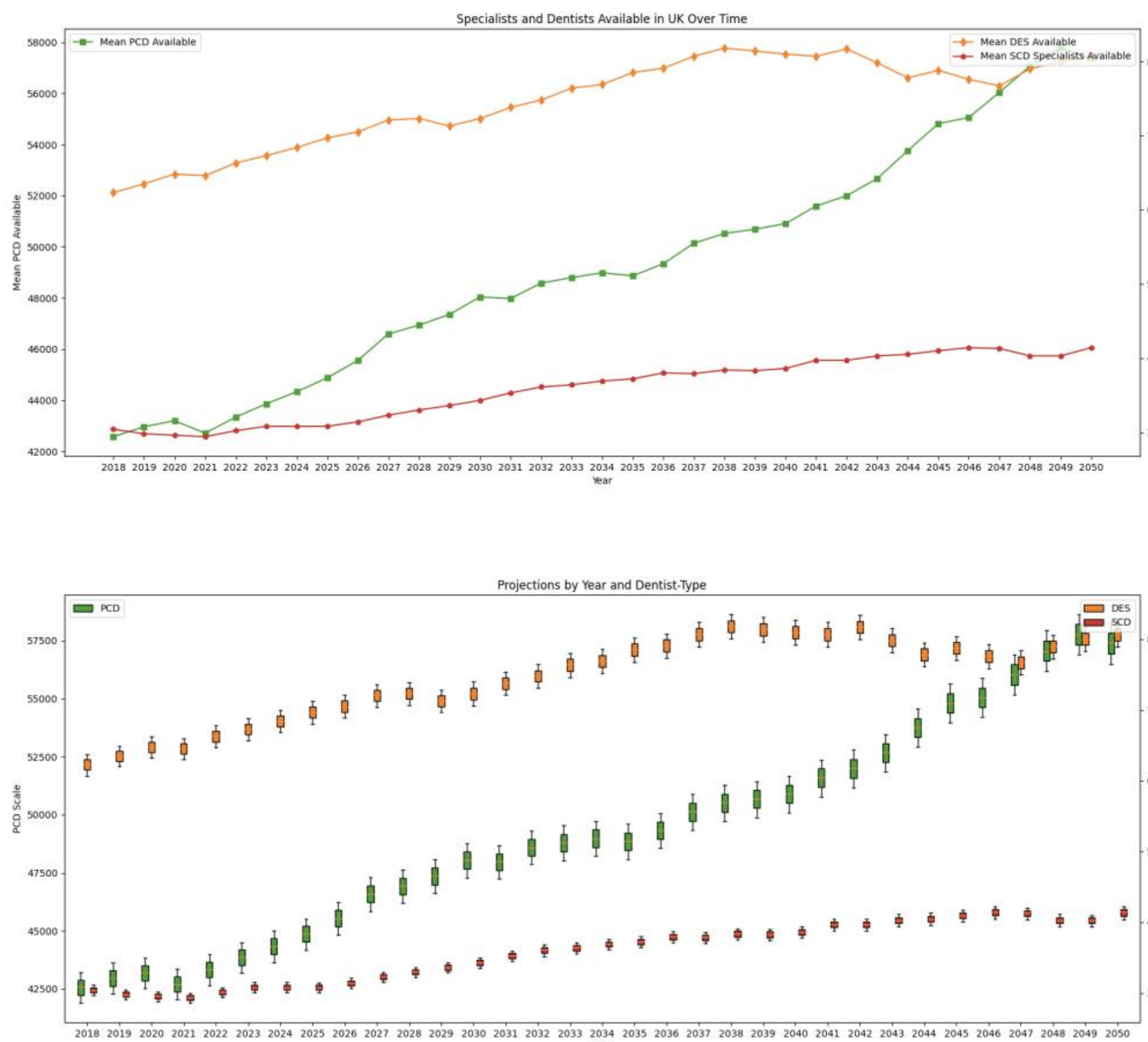


*Figure 8 Number of All types of Dentists Available Projections 2020-2050*

Description: The graphs above show 2020-2050 projections ranges and averages of the 3 types of dentists
Data Sources: ONS, GDC
Note:

## A4. Additional Figures

### Supply Tool Model Validation:

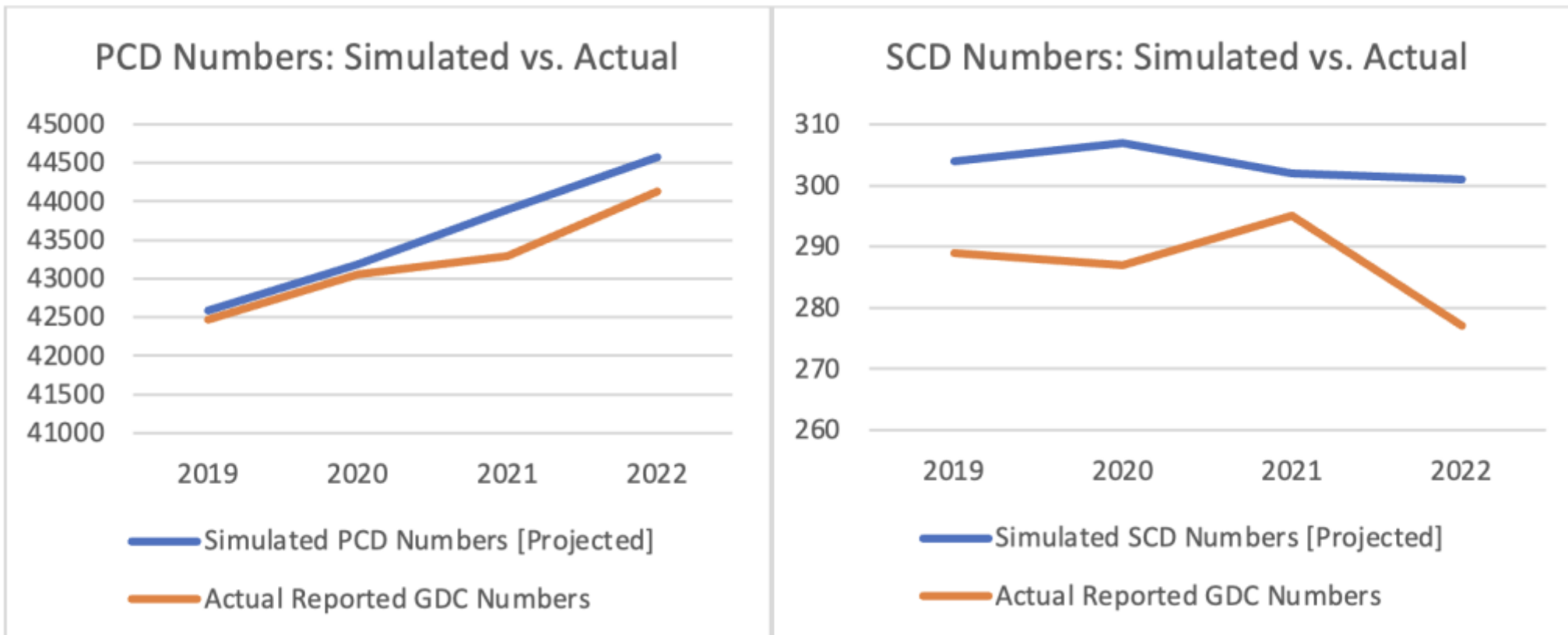


*Figure 9 Supply Tool Model Validation*

Description: Graphs above show the model validation - the simulated projected numbers of dentists from the tool vs the actual reported GDC numbers for the same years
Data Sources: GDC
Note:

### Overall Picture of Population Need vs. Supply for all Dentists in the UK:

| Workforce Category | % Allocation | | | | | | | | | | |
|---|---|---|---|---|---|---|---|---|---|---|---|
| Primary Care Dentists (PCD) | Share of total demand | 55% | 55% | 55% | 55% | 55% | 55% | 55% | 55% | 55% | 55% |
| | Patients seen (millions) Demand Met | 7.5 | 7.5 | 7.5 | 7.5 | 7.5 | 7.5 | 7.5 | 7.5 | 7.5 | 7.5 |
| Dentists with Enhanced Skills (DES) | % of moderate / complex need | 0% | 5% | 10% | 15% | 20% | 25% | 30% | 35% | 40% | 45% |
| | Patients seen (millions) | 0.00 | 0.27 | 0.53 | 0.80 | 1.07 | 1.33 | 1.60 | 1.86 | 2.13 | 2.39 |
| SCD Specialists | % of moderate / complex need | 45% | 40% | 35% | 30% | 25% | 20% | 15% | 10% | 5% | 0% |
| | Patients seen (millions) | 1.97 | 1.75 | 1.53 | 1.31 | 1.09 | 0.87 | 0.66 | 0.44 | 0.22 | 0.00 |

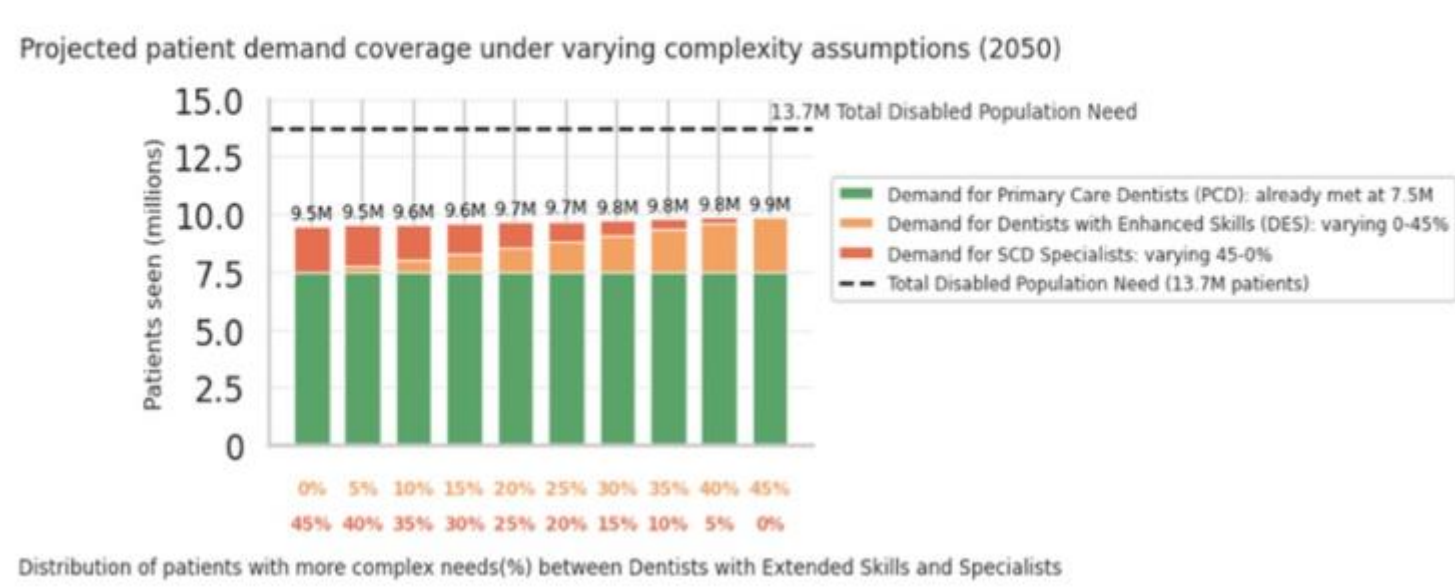


*Figure 10 Overall Picture of Population Need vs. Supply for all Dentists in the UK*

Description: The stacked bars illustrate the number of patients (in millions) who could be managed by each workforce group under different assumptions about case complexity. Primary Care Dentists (green) consistently meet demand for simple needs (7.5 million patients), while Dentists with Enhanced Skills (orange) and Specialists in Special Care Dentistry (red) together manage patients with moderate and complex needs. The dashed line represents the total projected disabled population need of 13.7 million patients by 2050.
Data Sources: GDC, ONS, Bannister's Paper
Note:

**Need vs. Supply Projections 2020-2050 for Primary care dentists, Dentists with Extended Skills, and SCD Specialists assuming 'Disabled; limited a lot' population split evenly (50:50) between moderate and complex needs (DES vs. SCD specialists)**

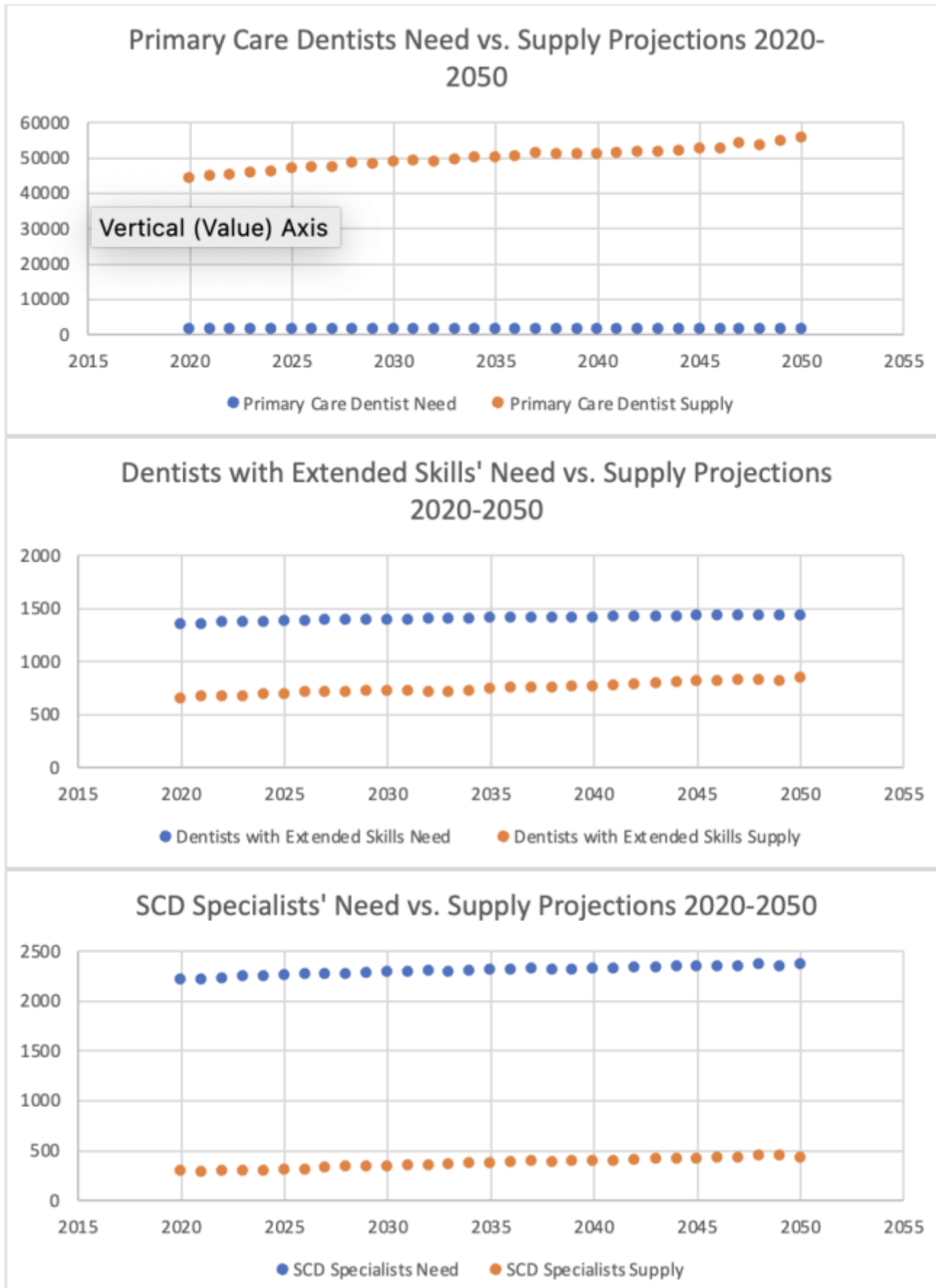


*Figure 11 Current Projected Need of all types of Dentists against respective Current Workforce Supply*

| |
|---|
| Description: Need vs. Supply Projections 2020-2050 for PCDs, DES', and SCD Specialists assuming 'Disabled; limited a lot' population split 50:50 between moderate and complex needs<br>Data Sources: ONS, GDC<br>Note: |

**OVERALL CURRENT SCD PICTURE – NEED ALL (100% - 10% Complex); BASELINE SCD SUPPLY (10 additional trainees/year)**

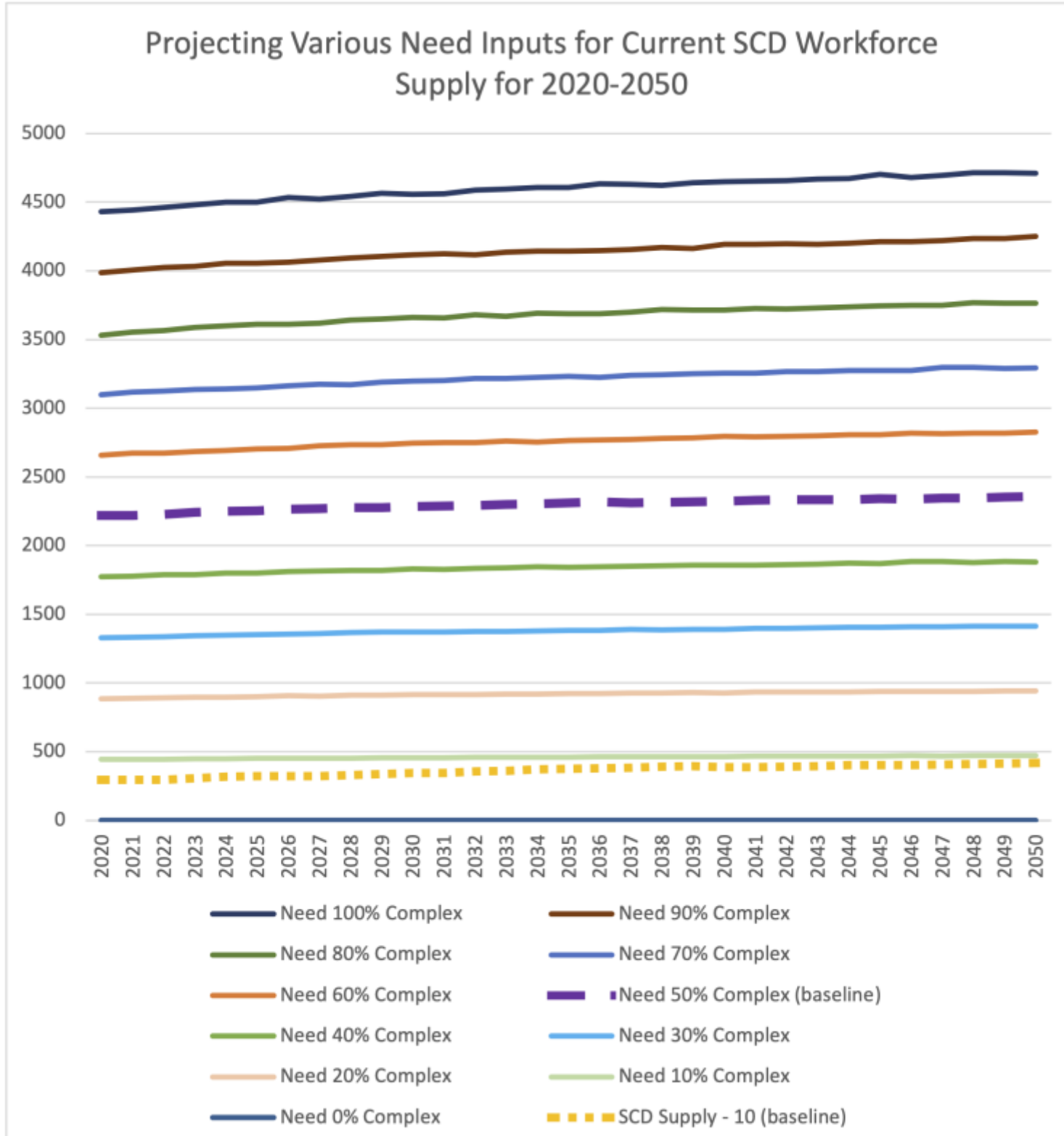


*Figure 12 – Allocating various proportions of "Disabled; limited a lot" population towards the complex needs category against the current SCD workforce yearly supply of 10 trainees/year*

Description: Allocating various proportions of "Disabled; limited a lot" population towards the complex needs category against the current SCD workforce yearly supply of 10 trainees/year
Data Sources: GDC, ONS
Note:

**GROWING SPECIALIST SDC WORKFORCE TO MEET NEED**

Allocating various proportions of the "disabled; limited a lot" population to have complex needs only

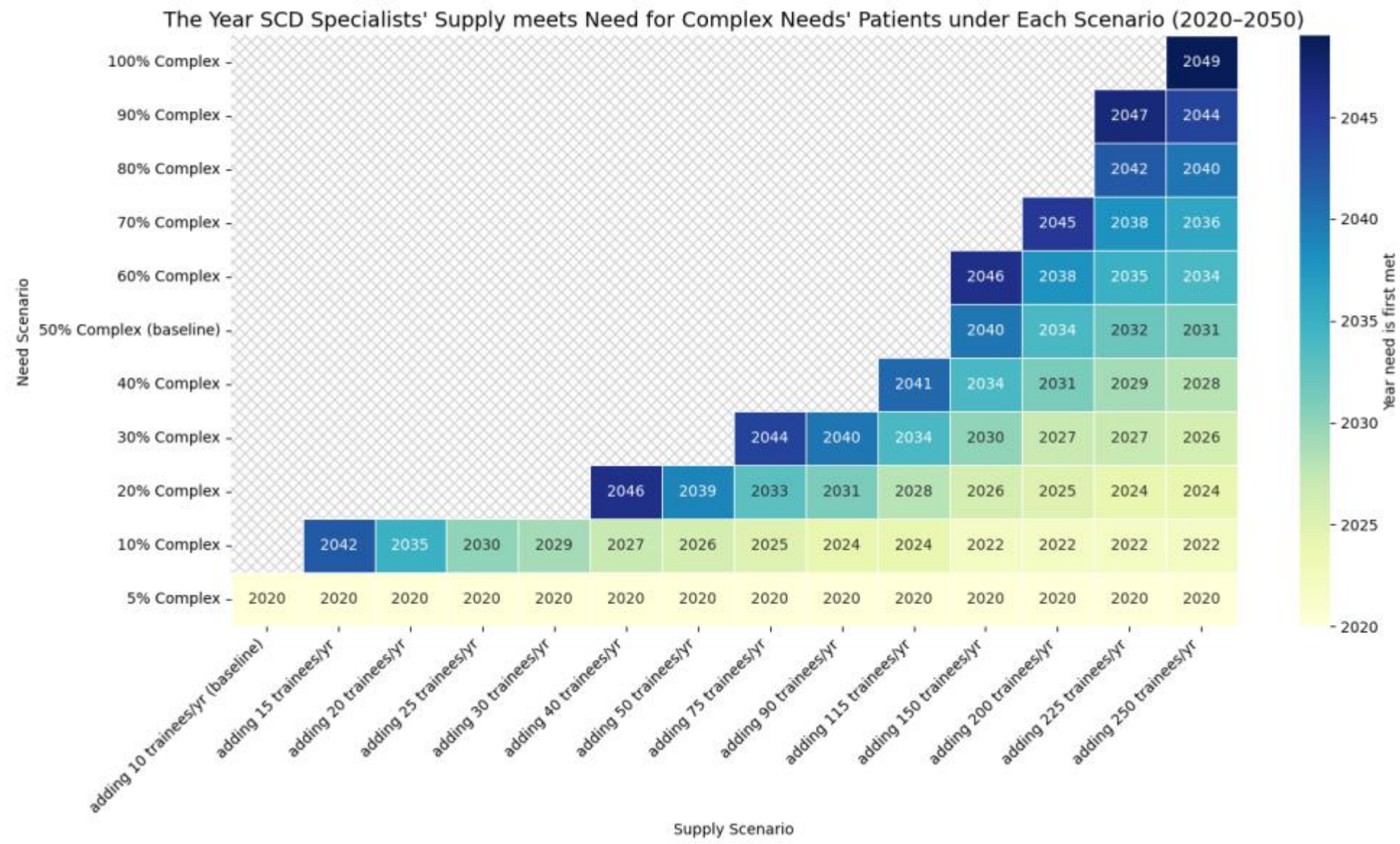


*Figure 13 – Various Projections of Number of SCD Specialists Available to meet Complex Patients' Need by 2050*

Description: Heatmap showing the first year in which projected SCD supply meets or exceeds need under varying assumptions for the proportion of complex-care patients (5–100%) and different specialist training intake scenarios (baseline and incremental trainee additions). Lighter shades indicate earlier years when workforce sufficiency is achieved, whereas darker shades correspond to later years. Cross-hatched cells represent scenarios where need is not met by 2050
Data Sources: GDC, ONS
Note:

## OVERALL CURRENT DENTISTS WITH EXTENDED SKILLS (DES) PICTURE – NEED ALL (100% - 10% MODERATE)

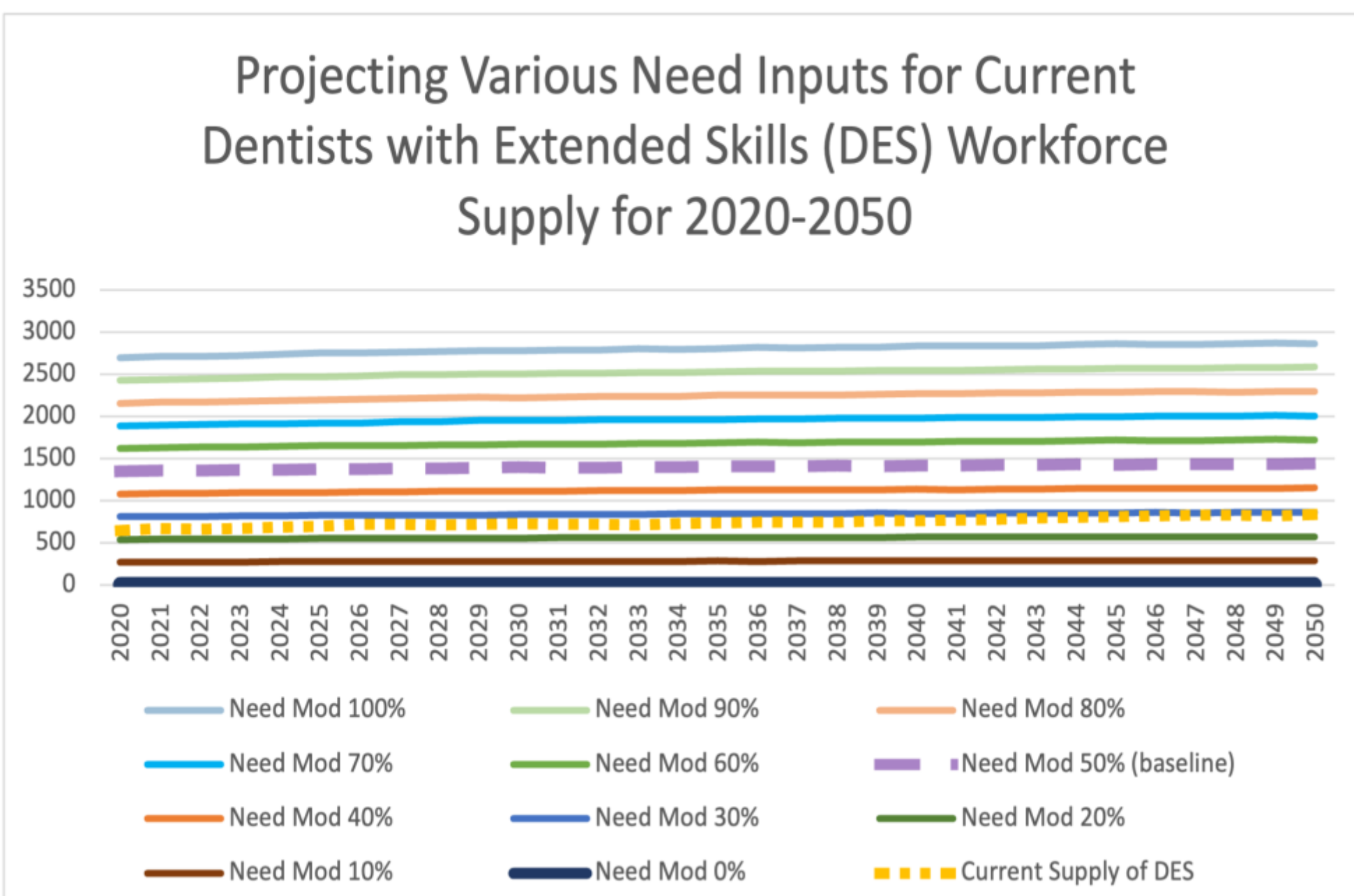


*Figure 15 – Projected Need of DES for various scenarios against Current DES Workforce Supply*

Description: Allocating various proportions of "Disabled; limited a lot" population towards the moderate needs category against the current DES workforce supply
Data Sources: GDC, ONS
Note:

## Building extra Dentists with Extended Skills (DES) Workforce to meet the Moderate Needs

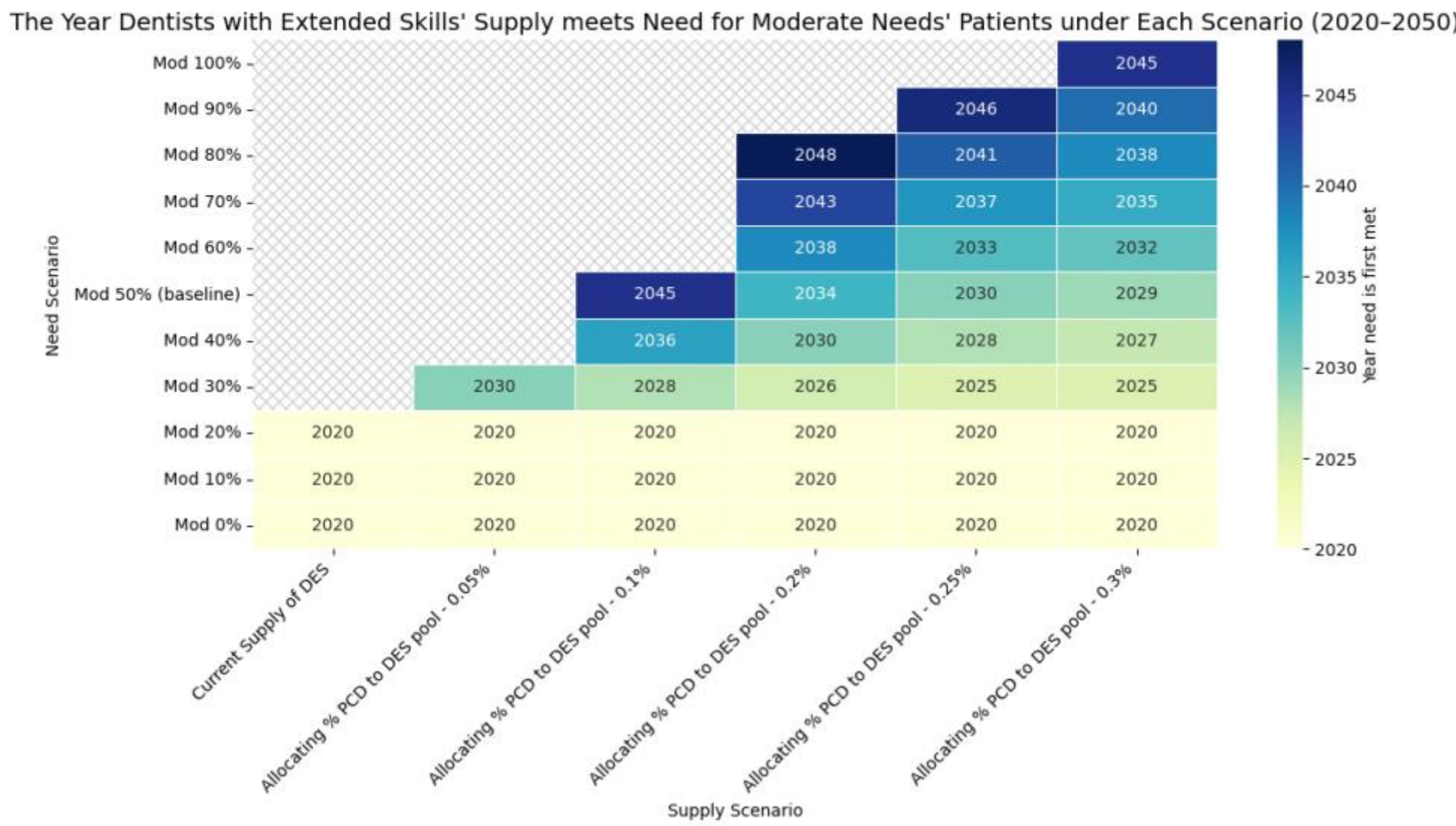


*Figure 16 – Various Projections of Number of DES Available to meet Moderate Patients' Need by 2050*

Description: Heatmap illustrating the first year in which supply for moderate complex dental cases meets projected need across baseline and percentage reallocation scenarios from the Primary Care Dentistry pool to the DES pool. Lighter shades indicate earlier years when workforce sufficiency is achieved, whereas darker shades correspond to later years. Cross-hatched cells represent scenarios where need is not met by 2050
Data Sources: GDC, ONS

## A5. References


Alzheimer Scotland. 2025. What is dementia? Available at: https://www.alzscot.org/what-is-dementia/ [Accessed: 19 September 2025].

Alzheimer's Society. 2023. Briefing – Northern Ireland budget 2023/24. London: Alzheimer's Society. Available at: https://www.alzheimers.org.uk/sites/default/files/2023-05/230510-alzheimers-society-briefing-northern-ireland-budget.pdf [Accessed: 19 September 2025].

Ashtari, P. et al. 2023. Dental careers: 2022 update. *Faculty Dental Journal* 14(2), pp. 46–53.

Bannister, C. et al. 2023. Time to complete contemporary dental procedures – estimates from a cross-sectional survey of the dental team.

Borreani, E. et al. 2008. Minimising barriers to dental care in older people. *BMC Oral Health* 8(1).

British Society for Disability and Oral Health (BSDH). (2018). Guidelines for Oral Health Care for People with Special Needs.

Department for Work and Pensions 2023. Family Resources Survey: Financial year 2021 to 2022 [Online] Available at: https://www.gov.uk/government/statistics/family-resources-survey-financial-year-2021-to-2022 [Accessed: 5 July 2023].

Gallagher, J. et al. 2023. Dental Public Health Consultant Workforce Capacity & Capability: Review to Action. *BASCD*.

Gallagher, J.E. and Fiske, J. 2007. Special care dentistry: A professional challenge. *British Dental Journal* 202(10), pp. 619–629.

General Dental Council. 2024. Registration Statistical Report 2024. London: General Dental Council. Available at: https://www.gdc-uk.org/docs/default-source/reports-and-publications/gdc-registration-statistics-report-2024.pdf [Accessed: 19 September 2025].

General Dental Council 2023. Registration statistical report 2022 - General Dental Council [Online] Available at: https://www.gdc-uk.org/docs/default-source/annual-reports/gdc_registration-statistical-report-22-23-v3_a.pdf?sfvrsn=80d57c7c_11 [Accessed: 7 July 2023].

Lewis, J. 2023. The cap on medical and dental student numbers in the UK [Online] Available at: https://researchbriefings.files.parliament.uk/documents/CBP-9735/CBP-9735.pdf [Accessed: 4 August 2023].

Marmot, M., Allen, J., Goldblatt, P., Boyce, T., McNeish, D., Grady, M., & Geddes, I. (2010). Fair Society, Healthy Lives: The Marmot Review. London: UCL Institute of Health Equity.

Morgan, M. Z., Monaghan, N. P., & Godson, J. H. (2018). Inequalities in dental care provision: The association between community factors and dental practices in England. Primary Dental Care, 25(1), 15-21.

Nash, A. 2020. Subnational population projections for England: 2018-based [Online] Available at: https://www.ons.gov.uk/peoplepopulationandcommunity/populationandmigration/populationprojections/bulletins/subnationalpopulationprojectionsforengland/2018based [Accessed: 2 July 2023].

Newton, J.T. and Bower, E.J. 2005. The Social Determinants of Oral Health: New Approaches to conceptualizing and researching complex causal networks. *Community Dentistry and Oral Epidemiology* 33(1), pp. 25–34

NHS Digital. 2023**.** Dementia surveillance data, England. [Dataset]. Available at: https://digital.nhs.uk/data-and-information/publications/statistical/dementia-surveillance-data-2023.csv [Accessed: 7 July 2023].

NHS England. 2022. Special care dentistry: clinical standard. London: NHS England. Available at: https://www.england.nhs.uk/wp-content/uploads/2022/10/B1641-clinical-standard-special-care-dentistry.pdf [Accessed: 4 November 2025].

NHS England 2014. Improving Dental Care and Oral Health – A Call to Action [Online] Available at: https://www.england.nhs.uk/2014/02/improve-dental-cta/ [Accessed: 22 August 2023].

NHS England, Chief Dental Officer Team 2015. Guides for commissioning dental specialties special care dentistry [Online] Available at: https://www.england.nhs.uk/commissioning/wp-content/uploads/sites/12/2015/09/guid-comms-specl-care-dentstry.pdf [Accessed: 5 July 2023].

NHS England (Workforce, Training & Education) 2023. Distribution of Special Care Dentistry Training Posts. National 'Dental Education Reform Programme' (DERP), pp. 1–10.

NICE 2016. Overview: Oral health for adults in care homes: Guidance [Online] Available at: https://www.nice.org.uk/guidance/ng48 [Accessed: 20 August 2023].

NISRA 2023. Northern Ireland census 2021 - MS-D02: Long-term health problem or disability by Broad Age bands [Online] Available at: https://statistics.ukdataservice.ac.uk/dataset/northern-ireland-census-2021-ms-d02-long-term-health-problem-or-disability-by-broad-age-bands [Accessed: 5 July 2023].

Nitschke, I., & Muller, F. (2004). The impact of oral health on the quality of life in the elderly. Oral Health & Preventive Dentistry, 2 Suppl 1, 271-275.

Nitschke, I., & Radbruch, L. (2020). The need for special care dentistry education in Europe. European Journal of Dental Education, 24(1), 17-22.

Office for Health Improvement and Disparities 2021. Delivering Better Oral Health: An evidence-based toolkit for prevention [Online] Available at: https://www.gov.uk/government/publications/delivering-better-oral-health-an-evidence-based-toolkit-for-prevention [Accessed: 2 October 2023].

Older People's Commissioner for Wales. 2016. Dementia: more than just memory loss – the experiences of people living with dementia and those who care for them. Cardiff: Older People's Commissioner for Wales. Available at: https://olderpeoplewales.com/en/publications/dementia-more-than-just-memory-loss.aspx [Accessed: 19 September 2025].

ONS 2022a. Principal projection - Wales population in age groups [Online] Available at: https://www.ons.gov.uk/peoplepopulationandcommunity/populationandmigration/populationprojections/datasets/tablea25principalprojectionwalespopulationinagegroups [Accessed: 1 July 2023].

ONS 2022b. Principal projection - England population in age groups [Online] Available at: https://www.ons.gov.uk/peoplepopulationandcommunity/populationandmigration/populationprojections/datasets/tablea24principalprojectionenglandpopulationinagegroups [Accessed: 1 July 2023].

ONS 2022c. Principal projection - Northern Ireland population in age groups [Online] Available at: https://www.ons.gov.uk/peoplepopulationandcommunity/populationandmigration/populationprojections/datasets/tablea27principalprojectionnorthernirelandpopulationinagegroups [Accessed: 1 July 2023].

ONS 2022d. Principal projection - Scotland population in age groups [Online] Available at: https://www.ons.gov.uk/peoplepopulationandcommunity/populationandmigration/pop

ulationprojections/datasets/tablea26principalprojectionscotlandpopulationinagegroups [Accessed: 1 July 2023].

ONS 2022e. Principal projection - UK population in age groups [Online] Available at: https://www.ons.gov.uk/peoplepopulationandcommunity/populationandmigration/populationprojections/datasets/tablea21principalprojectionukpopulationinagegroups [Accessed: 1 July 2023].

ONS 2023. Disability in England and Wales, 2021 [Online] Available at: https://www.ons.gov.uk/peoplepopulationandcommunity/healthandsocialcare/disability/datasets/disabilityinenglandandwales2021 [Accessed: 1 July 2023].

Park, N. 2022a. Population estimates for the UK, England, Wales, Scotland and Northern Ireland: Mid-2021 [Online] Available at: https://www.ons.gov.uk/peoplepopulationandcommunity/populationandmigration/populationestimates/bulletins/annualmidyearpopulationestimates/mid2021 [Accessed: 1 July 2023].

Park, N. 2022b. United Kingdom population mid-year estimate [Online] Available at: https://www.ons.gov.uk/peoplepopulationandcommunity/populationandmigration/populationestimates/timeseries/ukpop/pop [Accessed: 1 July 2023].

Patel, J. et al. 2023. *A Survey of the Special Care Specialist Workforce and its Speciality trainees*.
Scotland's Census 2014. 2011 census [Online] Available at: https://www.scotlandscensus.gov.uk/about/2011-census/ [Accessed: 5 July 2023].

Sharpling, J. and Gallagher, J.E. 2017. Specialties: Relevance and reform in the UK. *Faculty Dental Journal* 8(1), pp. 10–19.

Steele, J.G. et al. 2012. Adult Dental Health Survey 2009: Transformations in British Oral Health 1968–2009. *British Dental Journal* 213(10), pp. 523–527.

WHO 2022. United Kingdom of Great Britain and Northern Ireland Data [Online] Available at: https://data.who.int/countries/826 [Accessed: 26 August 2023].

Woolley, S. and Lau, W. 2021. Ten Years on: An online questionnaire evaluation of the UK special care dentistry specialist workforce. *British Dental Journal*.
